# Location-Dependent DNA Methylation Signatures in a Clonal Invasive Crayfish

**DOI:** 10.3389/fcell.2021.794506

**Published:** 2021-12-09

**Authors:** Sina Tönges, Geetha Venkatesh, Ranja Andriantsoa, Katharina Hanna, Fanny Gatzmann, Günter Raddatz, Vitor Coutinho Carneiro, Frank Lyko

**Affiliations:** Division of Epigenetics, DKFZ-ZMBH Alliance, German Cancer Research Center (DKFZ), Heidelberg, Germany

**Keywords:** marbled crayfish, environmental adaptation, DNA methylation, epigenetic signature, bisulfite sequencing

## Abstract

DNA methylation is an important epigenetic modification that has been repeatedly implied in organismal adaptation. However, many previous studies that have linked DNA methylation patterns to environmental parameters have been limited by confounding factors, such as cell-type heterogeneity and genetic variation. In this study, we analyzed DNA methylation variation in marbled crayfish, a clonal and invasive freshwater crayfish that is characterized by a largely tissue-invariant methylome and negligible genetic variation. Using a capture-based subgenome bisulfite sequencing approach that covers a small, variably methylated portion of the marbled crayfish genome, we identified specific and highly localized DNA methylation signatures for specimens from geographically and ecologically distinct wild populations. These results were replicated both biologically and technically by re-sampling at different time points and by using independent methodology. Finally, we show specific methylation signatures for laboratory animals and for laboratory animals that were reared at a lower temperature. Our results thus demonstrate the existence of context-dependent DNA methylation signatures in a clonal animal.

## Introduction

DNA methylation is a highly conserved epigenetic modification (Law and Jacobsen, 2010; Schubeler, 2015). Animal DNA methylation is found mainly in the CpG sequence context, with major differences in methylation patterning between species (Breiling and Lyko, 2015; Schubeler, 2015). Ubiquitous methylation patterns can be observed in vertebrate genomes, where 70–80% of all CpGs are methylated. Methylation levels in invertebrates are often lower than in vertebrates and are mainly found at specific genetic features like gene bodies or repeats (Breiling and Lyko, 2015; Schubeler, 2015).

It has been hypothesized that an important function of DNA methylation is to adapt genomes to changing environments (Jaenisch and Bird, 2003; Feil and Fraga, 2012). Pioneering work in this context has been performed in *Arabidopsis thaliana*, a globally distributed plant that is characterized by locally adapted phenotypes (Atwell et al., 2010). Methylation variation was suggested by an analysis of distinct lineages that were separated for 30 generations, but arose from the same ancestral lineage (Becker et al., 2011). However, later comparisons of genetic and epigenetic variances explained most of the methylation changes by genetic polymorphisms (Dubin et al., 2015), which are frequent in this species and define the large number of genetic ecotypes, rather than epigenetic ecotypes (Ferrero-Serrano and Assmann, 2019).

Also in animals, epigenetic changes have been interpreted to reflect responses to changing environments. For example, a recent study found methylation differences between salmon that were reared in artificial hatcheries and wild salmon (Le Luyer et al., 2017). While these methylation differences might explain the known fitness differences between the two environments, the study also noted the potential confounding effects of genetic polymorphisms (Le Luyer et al., 2017). Another prominent example is provided by the brown anole lizard (*Anolis sagrei*), where changes in methylation patterning were observed when lizards were exposed to a different habitat for 4 days (Hu et al., 2019). However, it could not be excluded that the observed changes were related to changes in the cell-type composition of the sampled organ (liver), which is important for the metabolism, thermoregulation, and immune function of lizards. Finally, promotor hypermethylation has been linked to transcriptional repression of eye-specific genes in cavefish (Gore et al., 2018). The study also showed that the injection of a DNA methylation inhibitor partially restored eye development, suggesting a functional role of epigenetic mechanisms in this context.

Adaptivity is also a crucial trait for invasive species, as it allows them to rapidly respond to newly colonized environments (Ghalambor et al., 2007; Fox et al., 2019). In invasive species, rapid adaptation often cannot be explained by the traditional selection of genetic variants, which requires longer timeframes (Carneiro and Lyko, 2020). Indeed, several studies have explored possible epigenetic mechanisms in this context. Examples are the mussel *Xenostrobus secures*, where global DNA hypomethylation was suggested to promote higher phenotypic plasticity (Ardura et al., 2017), or the whitefly (*Bemisia tabaci*), where the knockdown of a DNA methyltransferase resulted in a higher sensitivity to thermal changes (Dai et al., 2018).

It has been noted that important aspects in the design and interpretation of epigenome mapping studies are often not fully addressed (Lappalainen and Greally, 2017; Lea et al., 2017). For example, epigenetic patterns can be cell-type specific, but epigenetic profiles are often obtained from whole animals or bulk tissue. Furthermore, epigenetic effect sizes are often relatively small in ecological studies. Conclusive results thus require the design of sufficiently powered studies with relatively high sequencing depths and relatively large sample numbers. Finally, wild specimens from one species can have very heterogeneous genetic backgrounds, which can introduce a strong confounding effect in the analysis.

The marbled crayfish (*Procambarus virginalis*) represents an invasive species with a largely monoclonal genome (Gutekunst et al., 2018; Maiakovska et al., 2021), thus providing a unique opportunity to study rapid adaptation by epigenetic mechanisms with little or no influence of genetic alterations. Marbled crayfish emerged from a single animal in the German aquarium trade about 25 years ago (Scholtz et al., 2003; Lyko, 2017) and have been introduced into various freshwater systems by anthropogenic releases. The animals have formed numerous stable populations in a diverse set of habitats in different climate zones, and fundamentally different water bodies (Chucholl et al., 2012; Maiakovska et al., 2021). For example, in Madagascar, marbled crayfish colonized lentic as well as lotic water bodies, including rivers, lakes, ponds, swamps, rice fields, gravel pits, and drainage ditches and climatic zones from humid to sub-arid (Andriantsoa et al., 2019). This rapid colonization, in combination with near-monoclonality implies non-genetic mechanisms in the adaptation process.

DNA methylation has long been implied in the phenotypic variation of marbled crayfish (Vogt et al., 2008). More recently, a detailed characterization of the marbled crayfish methylome revealed considerable levels of CpG-specific methylation and a methylation landscape characterized by a mosaic pattern targeted to the gene bodies of housekeeping genes (Gatzmann et al., 2018). Interestingly, a comparative analysis of different tissues and samples from various animals and developmental stages established a relatively stable and mainly tissue-invariant methylation pattern (Gatzmann et al., 2018). While these findings provided a comprehensive characterization of the general features of the marbled crayfish methylome, the identification of context-dependent methylation changes was precluded by low sample numbers and low sequencing depth.

Our study reports the identification of population-specific DNA methylation signatures in marbled crayfish. To this end, we established a capture-based methylation assay targeting a subset of several hundred genes, which represent the variable portion of the marbled crayfish methylome. Data analysis from N = 48 specimens from four distinct habitats identified specific and highly localized methylation signatures. We also validated the results from the capture-based methylation analysis by re-sampling and by an independent methodology. Finally, we provide first evidence suggesting that methylation patterns can be altered in laboratory experiments.

## Materials and Methods

### Ethics Approval Statement

Animal collections were performed by approval of local fishery authorities in Germany and under research permits No. 58/19/MEDD/SG/DGF/DSAP/SCB.Re and No. 59/19/MEDD/SG/DGF/DSAP/SCB.Re in Madagascar. All laboratory experiments were performed by approval of the institutional animal welfare committee, in compliance with local standards and guidelines.

### Sampling and Laboratory Culture of Marbled Crayfish

Sampling for the initial analysis (capture-based bisulfite sequencing) was carried out between August and October 2017 in Germany and between October 2017 and March 2018 in Madagascar. Sampling for the validation experiments (targeted bisulfite sequencing) was carried out between March and May 2019 in Germany and Madagascar. Tissue samples were initially preserved in 100% ethanol and later stored at −80°C. Physicochemical water parameters were analyzed by Raiffeisen-Laborservice (Ormont, Germany).

Laboratory animals (total length at the start of the experiment: approx. 3 cm) were kept in 26 × 18 × 14 cm plastic containers and fed with aquarium feed. Tap water was used as the water source and replaced once a week. Water temperature was maintained at 20°C (unless stated otherwise).

### DNA Extraction

Genomic DNA was isolated and purified from abdominal muscle and hepatopancreas tissue using a Tissue Ruptor (Qiagen), followed by proteinase K digestion and isopropanol precipitation. The quality of isolated genomic DNA was assessed on a 2,200 TapeStation (Agilent).

### Library Preparation for Agilent Sure Select Methyl-Seq Assay

Library preparation was carried out as described in the SureSelectXT Methyl-Seq Target Enrichment System for Illumina Multiplexed Sequencing Protocol, Version D0, July 2015. Quality controls were performed, and sample concentrations were measured on a 2,200 TapeStation (Agilent). Multiplexed samples were submitted to the DKFZ High Throughput Sequencing core facility and sequenced on a HiSeq system (Illumina).

### DNA Methylation Analysis and Identification of Differentially Methylated Regions

A variance cutoff of >0.006 was applied to a previously published whole-genome bisulfite sequencing dataset (Gatzmann et al., 2018), identifying 846 genes. 149 of these were consistently methylated or unmethylated (mean ratio >0.8 or <0.2, respectively) and therefore excluded from further analysis, thus defining a core set of 697 variably methylated genes. Read pairs were quality trimmed and mapped to the core set using BSMAP (Xi and Li, 2009). Subsequently, the methylation ratio for each CpG site was calculated using the Python script provided with BSMAP. Only those CpG sites that were present in all the samples with a coverage of ≥5X were considered for further analysis. The average methylation level for each gene was calculated only if a gene had at least five CpG sites with ≥5X coverage. Furthermore, the genes that met the following criteria for methylation invariance were excluded from subsequent analysis: 1) genes that were in the bottom 10% in terms of methylation variance 2) genes with an average methylation level of <0.1 or >0.9, and 3) genes with more than 50% Ns in their sequence.

In order to identify tissue-specific differentially methylated genes, a Wilcoxon rank sum test was applied (hepatopancreas vs. abdominal muscle samples from Singlis and Reilingen) and the *p*-values were corrected for multiple testing using the Benjamini-Hochberg method. Likewise, to identify location-specific differentially methylated genes, a Kruskal-Wallis test was used, and the *p*-values were corrected for multiple testing using the Benjamini-Hochberg method. Additionally, dmrseq (Korthauer et al., 2019) was used with default parameters and a qval cutoff of 0.05 to identify tissue-specific and location-specific differentially methylated regions within our dataset. A principal component analysis (PCA) was performed based on the significantly differentially methylated genes. The PCA was carried out using the prcomp function in R and the PCA plots were generated using the R package ggfortify.

### Characterization of Variably Methylated Genes

Enrichment for housekeeping genes was analyzed by mapping marbled crayfish genes to protein sequences of human housekeeping genes using BLASTp, using an e-value cutoff of <10^−10^. *p*-values were calculated using a chi-square test. Enrichment for transposable elements (TEs) was analyzed by identifying TE annotation overlapping with gene body annotation and *p*-values were calculated using a chi-square test. Additionally, average gene body methylation levels were quantified for both the gene sets to examine if the core set was enriched with hypo- or hypermethylated genes. The methylation levels were computed based on published WGBS data from abdominal muscle (Gatzmann et al., 2018). Average gene expression was based on published RNA-seq data from abdominal muscle (Gatzmann et al., 2018). Gene expression levels (TPM values) were calculated using RSEM (Li and Dewey, 2011) and *p*-values were calculated using a *t*-test. The bar plots and stacked heatmaps were generated using the geom_bar function of ggplot2 in R. Network visualization of enriched GO molecular function terms of variably methylated genes was plotted using ShinyGO v0.0741 webtool.

### Validation of Differentially Methylated Regions

Genomic DNA was bisulfite converted by using the EZ DNA Methylation-Gold Kit (Zymo Research) following the manufacturer’s instructions. Target regions were PCR amplified using region-specific primers (Supplementary Table S1). PCR products were gel-purified using the QIAquick Gel Extraction Kit (Qiagen). Subsequently, samples were indexed using the Nextera XT index Kit v2 Set A (Illumina). The pooled library was sequenced on a MiSeqV2 system using a paired-end 150 bp nano protocol. Sequencing data was analyzed using BisAMP (Bormann et al., 2019).

## Results

### Habitat Profiles of Four Independent Marbled Crayfish Populations

**FIGURE 1:**
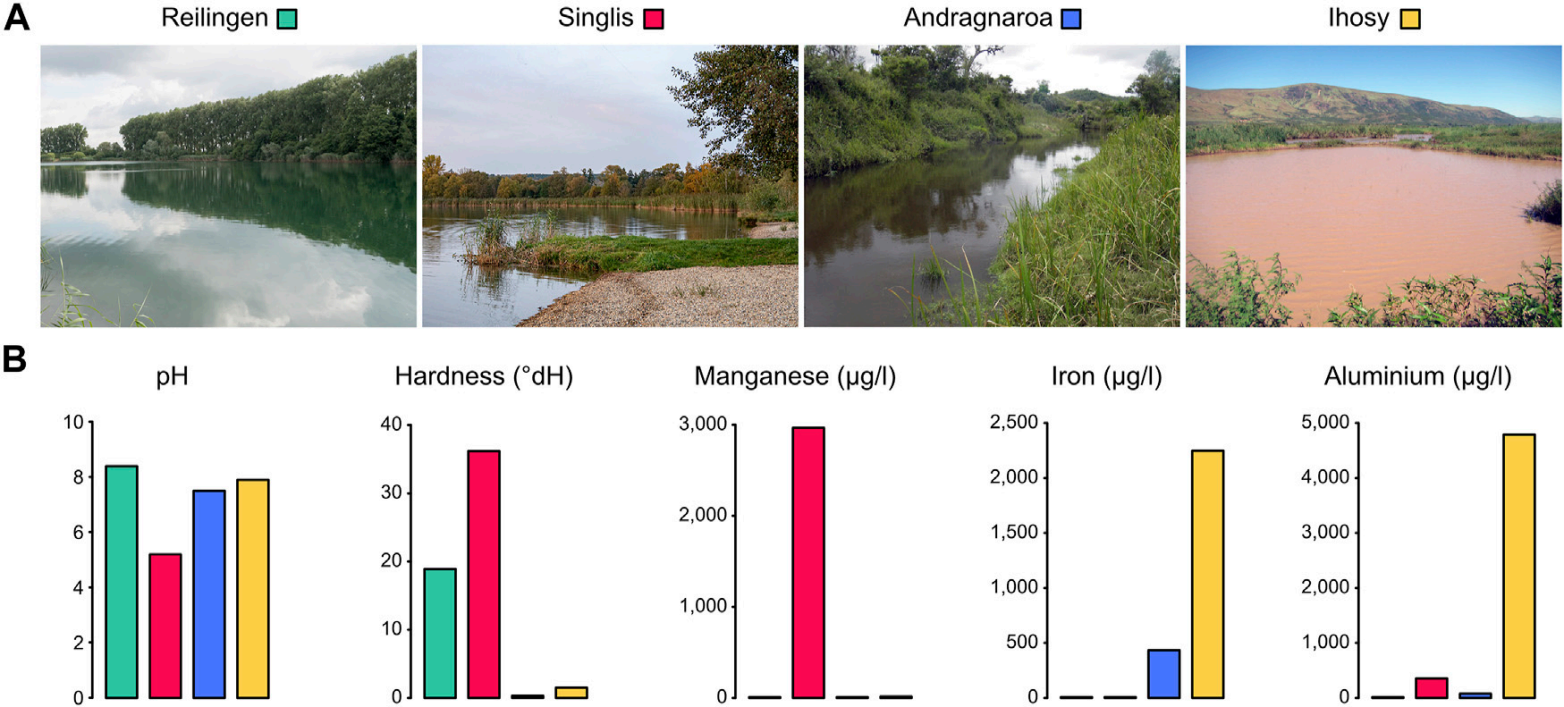
Marbled crayfish population habitats. **(A)** Habitat pictures of the four populations analyzed in this study **(B)** Selected water parameters of the four habitats analyzed.

Marbled crayfish are direct descendants from a single foundational specimen and were initially distributed through the global pet trade (Chucholl, 2015). Following anthropogenic releases, the animals have established numerous stable wild populations in a wide range of different habitats (Andriantsoa et al., 2019; Maiakovska et al., 2021). To identify location-specific DNA methylation signatures in marbled crayfish, we collected animals from four diverse populations (Figure 1A; Table 1). Reilingen (Germany) represents the type locality, a small eutrophic lake in an environmentally protected area. The Singlis (Germany) population is from a larger oligotrophic lake within a renaturalized brown coal mining area. The Andragnaroa (Madagascar) population is located in a river with soft mountain water flowing through a forest area at a relatively high altitude (1,156 m). Finally, the Ihosy (Madagascar) population is found in highly turbid water, with high levels of pollution from nearby mining activities. The analysis of physicochemical water parameters (Figure 1B) showed clean, slightly basic (pH 8.4) water in Reilingen and acidic (pH 5.2) water with high levels of Manganese (4,792 μg/L) in Singlis. The water in Andragnaroa showed particularly low hardness (0.3 dH), while the water in Ihosy was characterized by high levels of Aluminium (2,967 μg/L) and Iron (2,249 μg/L). Our study thus covers populations that inhabit four diverse habitats from different climatic zones and with different water parameters.

**TABLE 1:** Overview of marbled crayfish populations analyzed.

Site name	Coordinates	Type	Altitude (m)	Key features	Ground sediment	Associated vegetation and fauna
Reilingen (Germany)	N49°17,649′, E08°32,672′	lake	69	eutrophic lake	mud, sand	herbaceous grasses, macrophytes, algae, fish, insects, crayfish
Singlis (Germany)	N51°03.655′, E09°18.710′	lake	168	oligotrophic lake, acidic water	sand, pebbles	herbaceous grasses, insects
Andragnaroa (Madagascar)	S21°17.551′, E47°22.292′	river	1,156	slow-flowing mountain river	mud	herbaceous grasses, rice, fish, insects, crabs, crayfish
Ihosy (Madagascar)	S22°22.512′, E46°06.016′	river	711	slow-flowing, turbid, polluted river	mud	herbaceous grasses, fish, amphibians, molluscs, insects

### Identification of a Variably Methylated Gene Set

**FIGURE 2:**
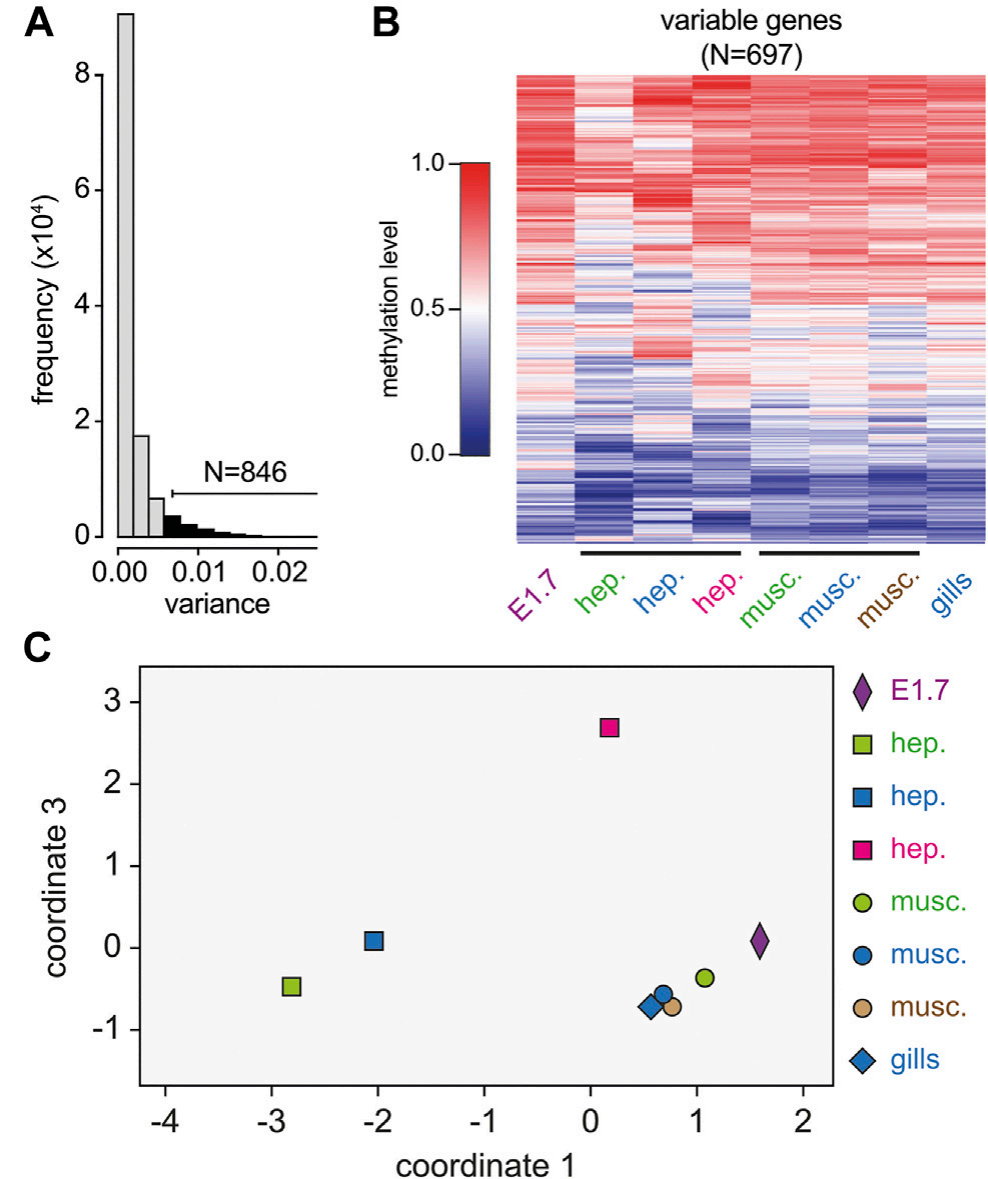
Identification and characterization of variably methylated genes by whole-genome bisulfite sequencing. **(A)** Density plot of methylation variance for the 12,244 genes with sufficient coverage in all eight samples. 846 of these genes had a methylation variance >0.06. **(B)** Heatmap showing average gene body methylation levels for the 697 variably methylated genes with a mean ratio >0.2 and <0.8 in eight independent samples (columns). Methylation levels are indicated on a scale from 0 (blue) to 1 (red). **(C)** Metric multi-dimensional scaling analysis of all eight samples based on the methylation levels of the 697 variably methylated genes. E1.7: embryonic stage 1.7, hep.: hepatopancreas, musc.: abdominal muscle. Colors indicate samples from individual specimens.

We have previously shown that DNA methylation in the marbled crayfish is targeted to gene bodies, and that DNA methylation patterns are largely stable and tissue-invariant (Gatzmann et al., 2018). However, a comparison of eight whole-genome bisulfite sequencing datasets from different animals, different tissues, and different developmental stages also identified a subset of genes that showed more variable methylation levels (Gatzmann et al., 2018). This was confirmed by systematic analyses of methylation variance (Figures 2A,B). Metric multidimensional analysis based on the methylation levels of a set of 697 variably methylated genes (see Materials and Methods for details) discriminated the hepatopancreas samples from the abdominal muscle samples (Figure 2C). This finding suggested the presence of subtle, previously unrecognized tissue-specific methylation patterns in marbled crayfish.

To determine the methylation patterns of these genes in a large number of samples and at high sequencing coverage, we developed a bead-based capture assay. Custom-made baits were designed to cover the entire coding sequences of the 697 pre-selected variably methylated genes. By hybridization of the samples to the baits, only the genes of interest were captured. After removal of unbound DNA, captured DNA was bisulfite converted and sequenced on a Hi-Seq platform. Subgenome capture was found to be both efficient and specific, providing a minimum of 10 million mapped reads per sample under stringent conditions (Supplementary Figure S1, Supplementary Table S2). Bisulfite conversion rates were usually >98% (Supplementary Table S2), which further underscores the quality of the dataset. We processed DNA samples from two different tissues: hepatopancreas, the main metabolic organ of crayfish and abdominal muscle. Hepatopancreas DNA was prepared from N = 48 animals (11–13 per location), while abdominal muscle DNA was prepared from a subset of the same animals (N = 27, 4–12 per location, see Supplementary Table S2).

In subsequent steps, genes with more than 50% Ns in their sequence were excluded from further analysis, which left 623 genes. Furthermore, only those CpG sites that were present in all the samples with a sequencing coverage of ≥5x were considered and average methylation levels were calculated only if a gene had ≥5 qualified CpG sites. These criteria were fulfilled for 463 genes. We also excluded invariant genes, i.e., genes that were in the bottom 10% for methylation variance as well as genes with an average methylation level <0.1 or >0.9, resulting in a core set of 361 variably methylated genes (Supplementary Table S3).

**FIGURE 3:**
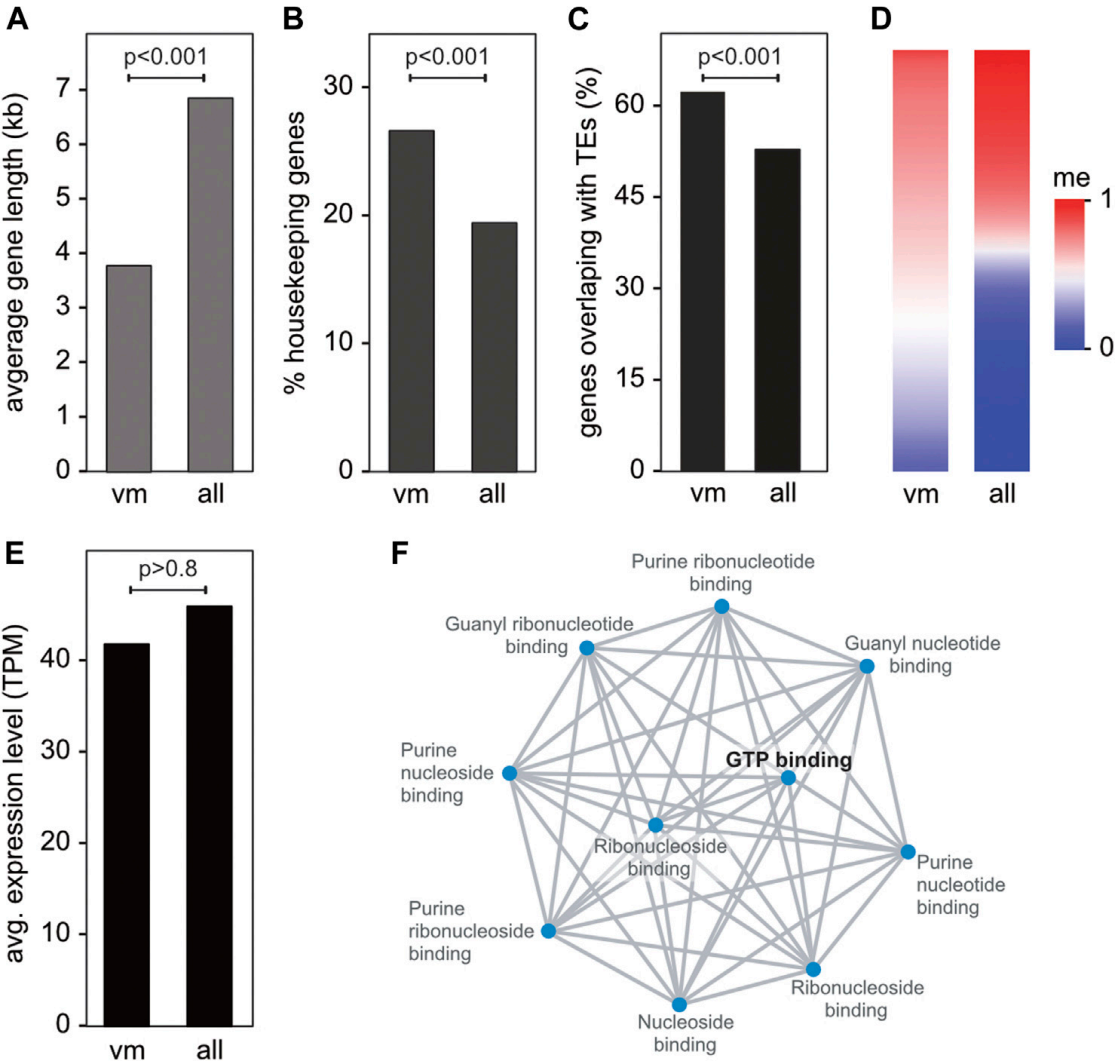
Characterization and functional annotation of the core set of 361 variably methylated genes. **(A)** Average gene length of variably methylated (vm) genes vs. all genes. **(B)** Percentage of housekeeping genes in the variably methylated gene set vs. all genes. **(C)** Percentage of variably methylated genes vs. all genes overlapping with transposable elements (TEs). **(D)** Heatmap depicting the average methylation of variably methylated genes and all genes. **(E)** Average gene expression of genes in the variably methylated gene set vs. all genes. **(F)** Network visualization of enriched GO molecular function terms of variably methylated genes. Adjusted *p*-values are <0.0002 for all terms.

Characterization of the 361 variably methylated genes showed that these genes were distinctly shorter (Figure 3A) and enriched for housekeeping gene functions (Figure 3B), when compared to all genes. Variably methylated gene also showed a moderate (but significant) enrichment for transposable elements (Figure 3C) and were often characterized by intermediate methylation levels (Figure 3D) and similar expression levels compared to the transcriptome average (Figure 3E). Gene ontology analysis of the annotated variably methylated genes revealed a significant enrichment of GTP-binding proteins (Figure 3F), which are known for regulating various cellular processes (Syrovatkina et al., 2016). In agreement with this notion, variably methylated genes (Supplementary Table S3) were often associated with transcription/translation regulation, response to stress, RNA metabolism, and immune response to pathogens.

### Context-Dependent DNA Methylation Patterns in Marbled Crayfish Populations

**FIGURE 4:**
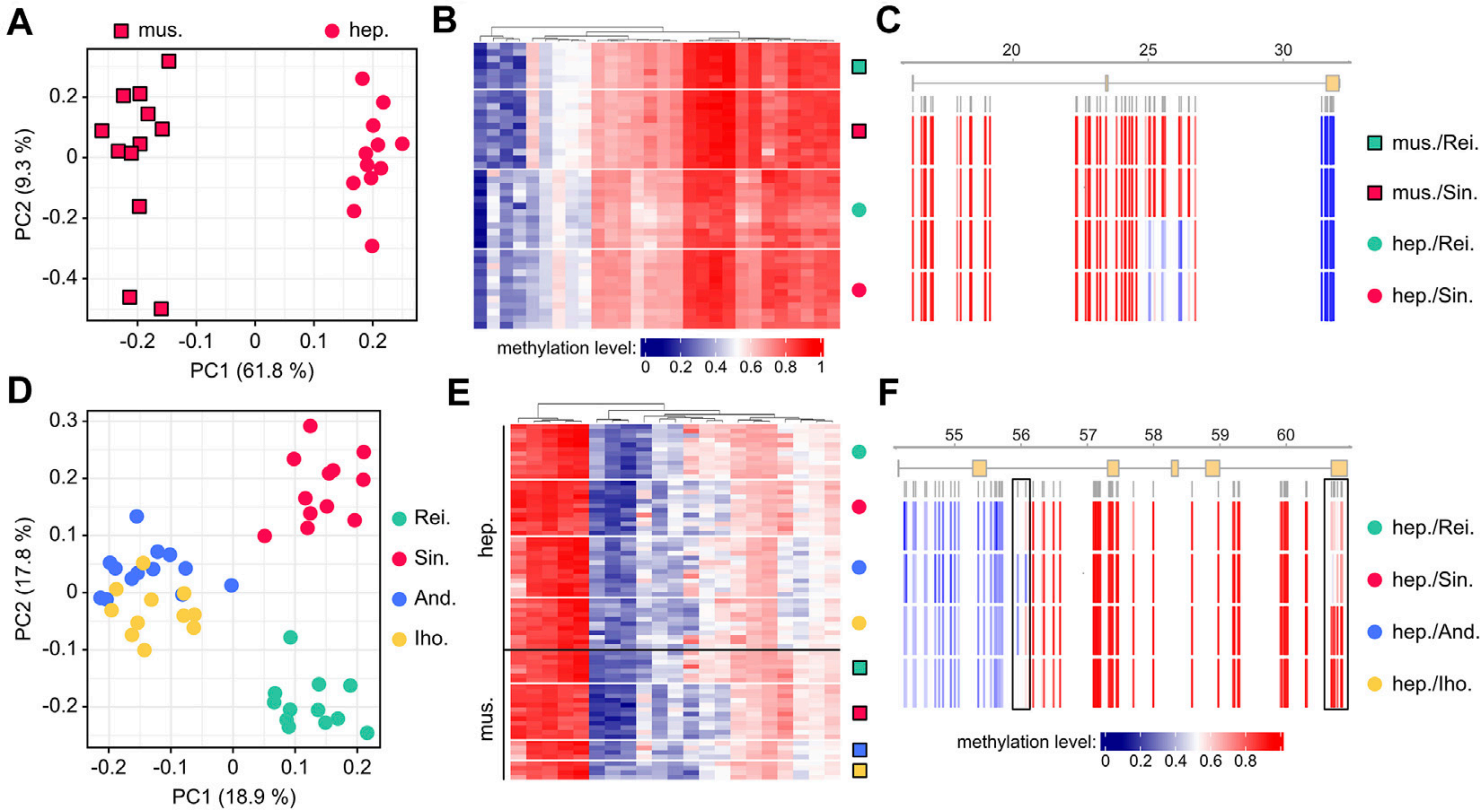
Location-dependent differential methylation in marbled crayfish populations. **(A)** Principal component analysis of individual abdominal muscle (mus, square symbols) and hepatopancreas (hep, circular symbols) samples from Singlis, based on the methylation levels of 56 genes with tissue-specific methylation differences. **(B)** Heatmap showing average methylation levels of 28 shared (Singlis and Reilingen) genes with tissue-specific methylation. Methylation levels are indicated on a scale from 0 (blue) to 1 (red) for Reilingen (green symbols) and Singlis (red symbols). **(C)** Average CpG methylation levels in a gene with tissue-specific methylation. **(D)** Principal component analysis of individual hepatopancreas samples from all locations, based on the methylation levels of 122 genes with location-specific methylation differences. **(E)** Heatmap showing average methylation levels of 21 shared (hepatopancreas and abdominal muscle) genes with location-specific methylation. Methylation levels are indicated on a scale from 0 (blue) to 1 (red), as in panel B. **(F)** Average CpG methylation levels in a gene with location-specific methylation. Boxes highlight differentially methylated regions.

We then used our core set of 361 variably methylated genes to identify tissue-specific methylation differences. To this end, we applied a Wilcoxon rank sum test for differential (*p* < 0.05 after Benjamini-Hochberg correction) methylation between hepatopancreas and abdominal muscle. For our largest dataset from a single location (Singlis, N = 24) this identified 56 genes that allowed a robust separation of the two tissues (Figure 4A). When the same approach was applied to the second-largest dataset (Reilingen, N = 19), it identified 35 differentially methylated genes (28 of which overlapped with Singlis) that again allowed a robust separation of the two tissues (Supplementary Figure S2). Tissue-specific methylation differences appeared relatively moderate for average gene methylation levels (Figure 4B), but more pronounced at the CpG level (Figure 4C). Of note, tissue-specific methylation differences were highly stable between the two populations (Figures 4B,C). These findings suggest the existence of localized tissue-specific methylation patterns in marbled crayfish.

To identify location-specific methylation differences, we applied a Kruskal-Wallis test for differential (*p* < 0.05 after Benjamini-Hochberg correction) methylation between the four sampled locations. For the larger hepatopancreas dataset (N = 48), this identified 122 genes that allowed a robust separation of the four locations (Figure 4D). A pairwise comparison between the Andragnaroa and Ihosy samples also achieved a clear separation between these two locations (Supplementary Figure S3A), which further confirmed the presence of location-specific methylation differences. When the same approach was applied to the smaller abdominal muscle dataset (N = 27), it identified 23 differentially methylated genes (21 of which overlapped with hepatopancreas) that again allowed a robust separation of the four locations (Supplementary Figure S3B). Similar to our findings for tissue-specific methylation, location-specific methylation differences appeared moderate for average gene methylation levels (Figure 4E) but more pronounced at the CpG level (Figure 4F). Also, location-specific methylation differences were highly stable between different tissues (Figures 4E,F). These findings strongly suggest the existence of defined location-specific methylation signatures in marbled crayfish populations.

### Validation of Context-Dependent Methylation Patterns

**FIGURE 5:**
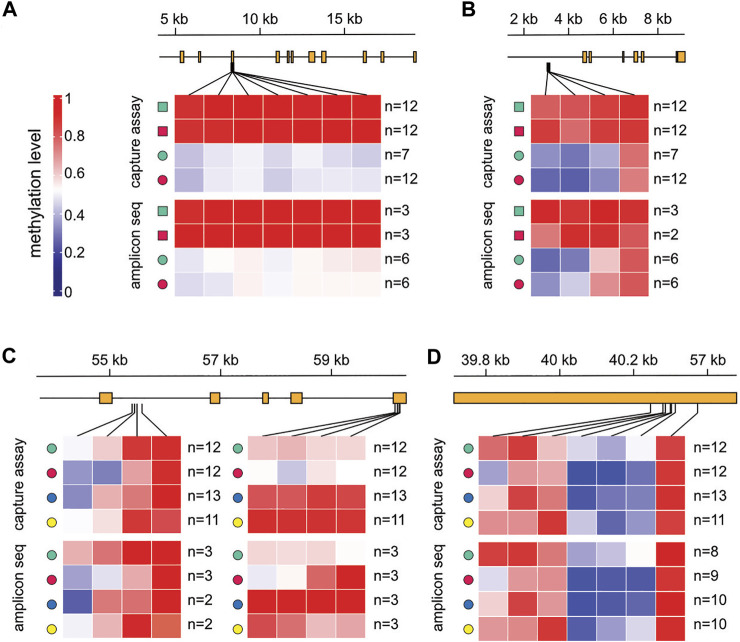
Validation of location-dependent differential methylation in marbled crayfish. Results are shown for capture-based sequencing and for the corresponding validation experiment with amplicon sequencing, for four different genomic regions. **(A)** scaffold 139,595: 4,000–19,145; **(B)** scaffold 220,173: 1,241–9,258; **(C)** scaffold 10,188:54,147–60,918; **(D)** scaffold 195,460: 39,668–40,474. Squares: abdominal muscle; circles: hepatopancreas; green: Reilingen; red: Singlis; blue: Andragnaroa; yellow: Ihosy.

To independently validate the tissue- and location-specific methylation patterns, we identified differentially methylated regions (DMRs, see Methods for details) within our set of 361 variably methylated genes and designed PCR assays for targeted bisulfite sequencing. This identified 37 tissue-specific DMRs and 68 location-specific DMRs (Supplementary Figure S4). In addition, we collected a fresh set of samples from all locations, one to 2 years after the initial sampling (see Methods for details). Deep sequencing of PCR amplicons provided direct confirmation of the capture-based subgenome sequencing results (Figure 5). We observed clear localized methylation differences between the tissues (Figure 5A) as well as between locations (Figure 5B), and the methylation ratios of individual CpGs were highly similar between the initial, capture-based dataset and the validation dataset obtained by targeted bisulfite sequencing. Interestingly, these results also indicate that location-specific methylation patterns among marbled crayfish populations are stable over time.

In additional controls, we addressed potential residual effects of genetic variants by intersecting the comprehensive set of known marbled crayfish SNVs, which includes SNVs from the populations analyzed in this study (Maiakovska et al., 2021), with the set of 361 variably methylated genes. This identified only five SNVs that overlapped with individual genes for location- or tissue-dependent separation. Furthermore, none of the five SNVs was located in the genes that were used for the validation experiments. These findings largely eliminate genetic variants as confounding factors in our analysis and suggest that the population-specific methylation signatures can be considered as purely epigenetic variants.

### Context-dependent Methylation Patterns in Laboratory Experiments

**FIGURE 6:**
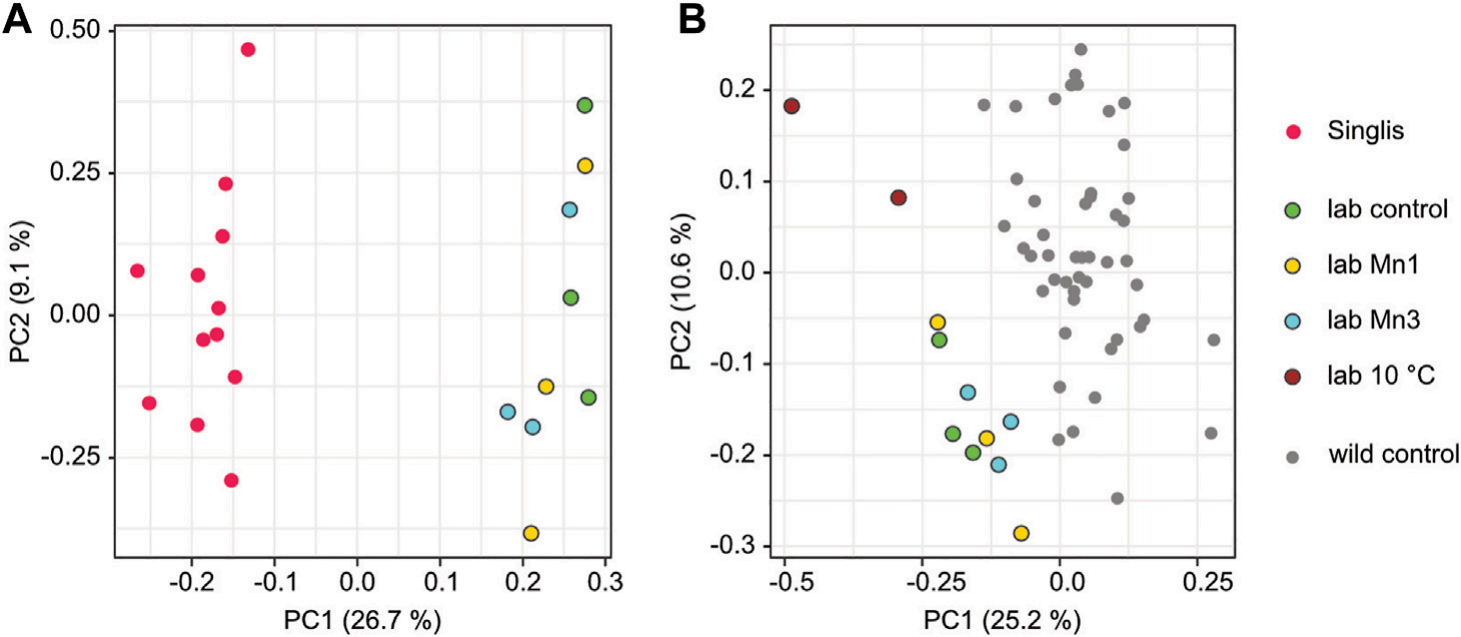
Experimental modeling of context-dependent methylation signatures. All samples for this analysis were taken from the hepatopancreas 6 months after the start of the experiment. Conditions are displayed in colors: green, control; dark red, 10°C; yellow, manganese 1 mg/L (Mn1); blue, manganese 3 mg/L (Mn3). **(A)** PCA based on the average methylation of the core set of 361 variably methylated genes, showing the separation of individual samples from Singliser See (red) and laboratory animals (green, yellow, and blue). **(B)** PCA based on the average methylation of the core set of 361 variably methylated genes, showing the separation of individual wild animals (gray) from laboratory animals (dark red, green, yellow, blue) and the separation of animals kept at low temperature (10°C, dark red) from other laboratory animals (green, yellow, blue).

Finally, we also set up laboratory experiments to model the impact of environmental changes on DNA methylation. In the first set of experiments, we kept animals in Manganese-supplemented water (1 mg/L or 3 mg/L) for 6 months to recapitulate the Manganese pollution in Singlis (Figure 1). Subsequent DNA methylation analysis of hepatopancreas samples using capture-based subgenome sequencing did not reveal a detectable effect of Manganese on the methylation pattern of these genes (Figure 6A). However, we observed a clear separation between the laboratory-reared animals and the wild-caught animals from Singlis (Figure 6A), suggesting that other factors (or combinations of factors) drive the epigenetic differences between laboratory-reared and wild-caught animals. Indeed, when we analyzed methylation patterns from two animals that were kept at 10°C for 6 months, we observed a robust separation from the other laboratory samples (kept at 20°C) and from the complete set of wild samples (Figure 6B). These results provide first evidence for environmentally induced methylation changes in marbled crayfish.

## Discussion

Adaptive evolution and adaptive phenotypic plasticity provide important responses to changing environmental conditions. Adaptive evolution usually occurs through gradual genetic changes over long evolutionary timeframes. However, this does not apply to the monoclonal marbled crayfish (Gutekunst et al., 2018). In light of the animal’s considerable adaptive potential (Andriantsoa et al., 2019), epigenetic mechanisms, such as DNA methylation, likely play an important role in phenotypic plasticity (Carneiro and Lyko, 2020).

We have previously shown that the marbled crayfish genome encodes a conserved and active DNA methylation system (Gatzmann et al., 2018). The analysis of several independent methylomes further revealed a relatively stable and tissue-invariant DNA methylation pattern (Gatzmann et al., 2018). Nevertheless, a subset of 697 genes showed more variable methylation patterns. We now used this geneset for a high-coverage subgenome sequencing approach of multiple animals to achieve sufficient statistical power for downstream analyses. Our results showed a robust separation of the different tissues and locations analyzed. Tissue-specific methylation patterns were consistent among collection sites, thus suggesting a tissue-related function of those genes. However, our previous analysis failed to detect any evidence for a correlation between DNA methylation and gene expression levels in marbled crayfish, but rather found a correlation between DNA methylation and gene expression variation (Gatzmann et al., 2018). The precise molecular function of DNA methylation in marbled crayfish thus remains to be elucidated.

It is also interesting to notice that tissue- and location-specific methylation differences were generally highly localized in the marbled crayfish genome. Similar observations have been made in the honey bee (*Apis mellifera*), where condition-specific methylation patterns were described in the brain (Kucharski and Maleszka, 2020). These localized methylation changes could conceivably influence several mechanisms, like interactions with chromatin, microRNAs, or the modulation of transcription factor binding (Ashby et al., 2016; Neri et al., 2017; Wojciechowski et al., 2018). Interestingly, our gene ontology analysis of variably methylated genes showed an enrichment of genes associated with GTP binding proteins. Whether GTP binding proteins play a role in adapting marbled crayfish to various environmental parameters will have to be determined in future studies.

Our results also show location-dependent methylation patterns are stably maintained in marbled crayfish. These signatures might represent neutral epialleles that are stably maintained in specific populations or adaptive epialleles that confer fitness advantages to local environmental parameters. The role of epigenetic mechanisms in rapid adaptation has been widely discussed in the literature (Schubeler, 2015; Verhoeven et al., 2016; Carneiro and Lyko, 2020), and the existence of epigenetic ecotypes has been claimed repeatedly. However, while genetic ecotypes are an established concept in ecological adaptation (Consortium, 2016), the existence of true epigenetic ecotypes, that are not confounded by genetic variation, has remained unclear. Genetic variation in marbled crayfish is extremely low (Gutekunst et al., 2018; Maiakovska et al., 2021), and was further excluded as a confounding factor in our analysis. As such, our study provides a conclusive and convincing example for location-specific epigenetic variation in animals.

Consistent with our observation of location-specific methylation signatures of wild animals, we also observed a distinct methylation signature for our laboratory colony. We further observed a detectable change in methylation patterns for animals that were kept at lower temperature for 6 months, but not for animals that were kept in Manganese-supplemented water. The reasons could be technical and/or biological. Our analysis was based on a small subset of genes, which may not display the methylation changes caused by manganese. Furthermore, the epigenetic response to different environmental stressors could potentially differ in time and magnitude. More resolved analyses of methylation changes that are triggered by a change in a specific environmental factor will help to better understand the relevance of DNA methylation for rapid adaptation. Functional approaches, such as CRISPR-mediated editing of DNA methyltransferase genes, will be important to directly determine the impact of DNA methylation on organismal adaptivity and plasticity.

## Data Availability

The datasets presented in this study are deposited in the NCBI repository, accession number PRJNA777002.
