# Supplementary material for: Location-Dependent DNA Methylation Signatures in a Clonal Invasive Crayfish
Source: Front Cell Dev Biol. 2021 Dec 9;9:794506. doi: 10.3389/fcell.2021.794506 (PMC8695926; doi:10.3389/fcell.2021.794506)
Supplement: Supplementary file 1 [file DataSheet1.docx]

Supplementary Material


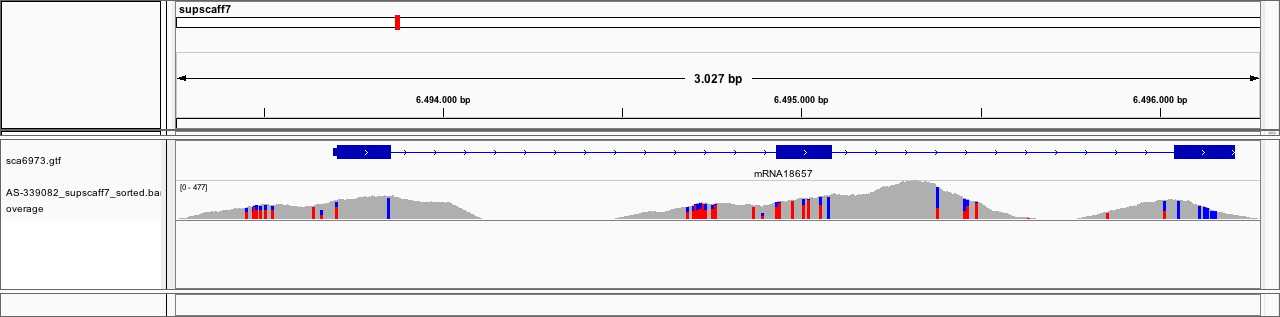


**Supplementary Figure S1.** Enrichment of sequencing reads in a target gene. Representative gene (3.027 bp) used for the capture-based assay. Capture design covered mainly exons (blue rectangles). Grey bars the enrichment of sequencing reads in the exon regions. Colored bars within the grey area show CpGs and their respective methylation levels (red: methylated fraction, blue: unmethylated fraction). The enrichment of the target regions provides proof for the specificity of the capture design.


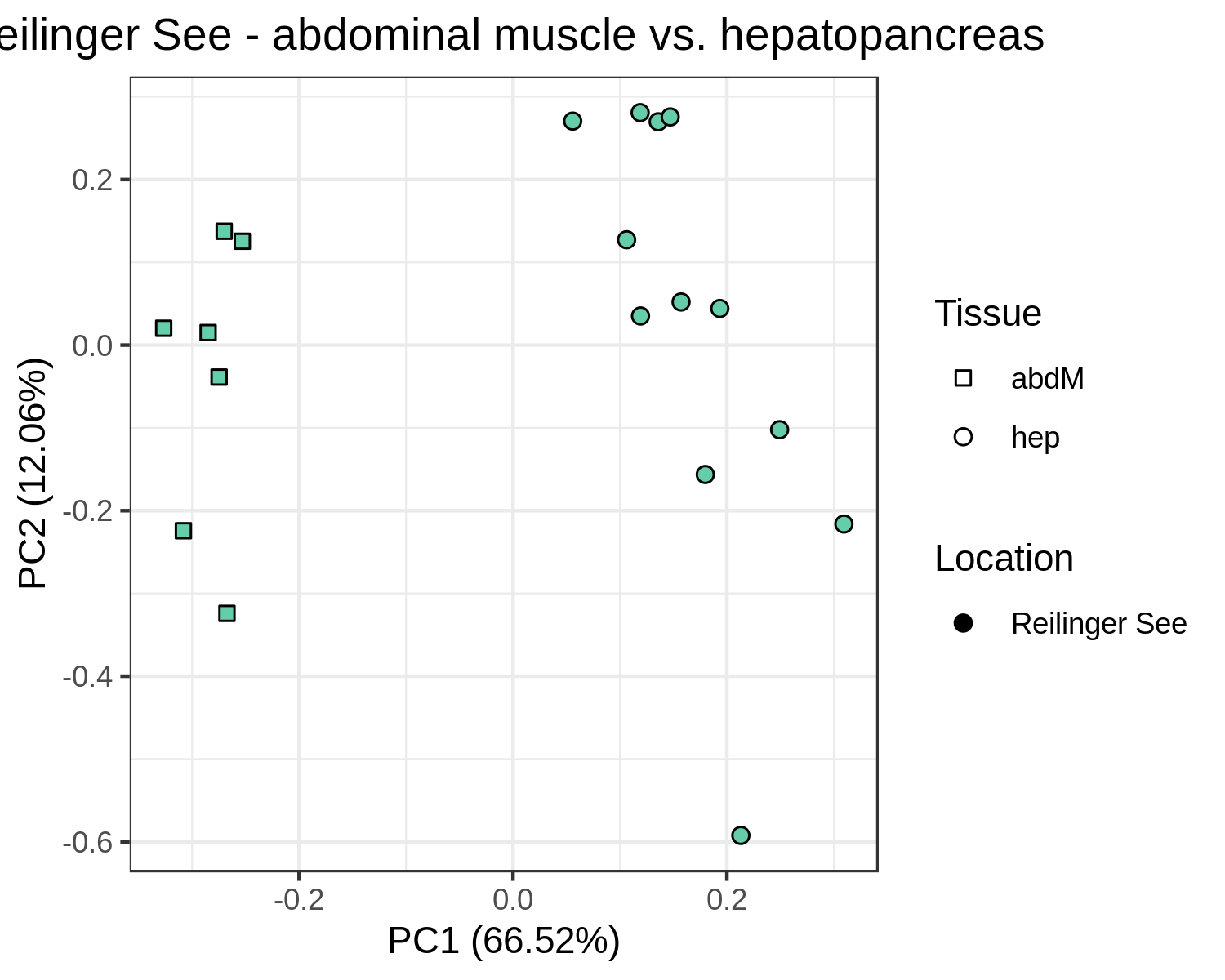

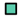

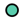


hep.

mus.

**Supplementary Figure S2.** Tissue-specific differential methylation in marbled crayfish populations. Principal component analysis of abdominal muscle (mus., square symbols) and hepatopancreas (hep., circular symbols) samples from Reilingen, based on the methylation levels of 35 genes with tissue-specific methylation differences.


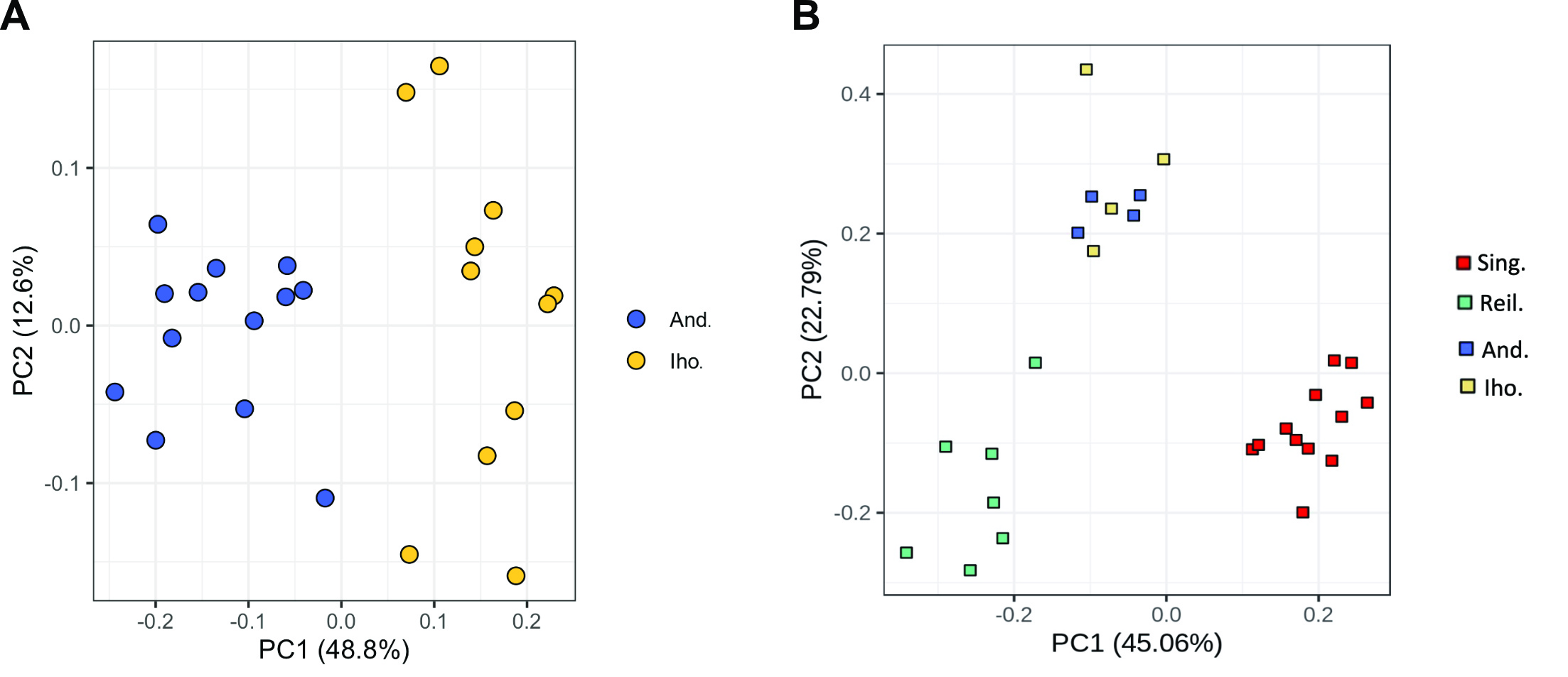


**Supplementary Figure S3.** Location-specific differential methylation in marbled crayfish populations. (**A**) Principal component analysis of hepatopancreas samples, based on the methylation levels of 53 genes that showed differential methylation between the two locations from Madagascar. **(B)** Principal component analysis of abdominal muscle samples from all analyzed locations, based on the methylation levels of 23 genes with location-specific methylation differences.

**
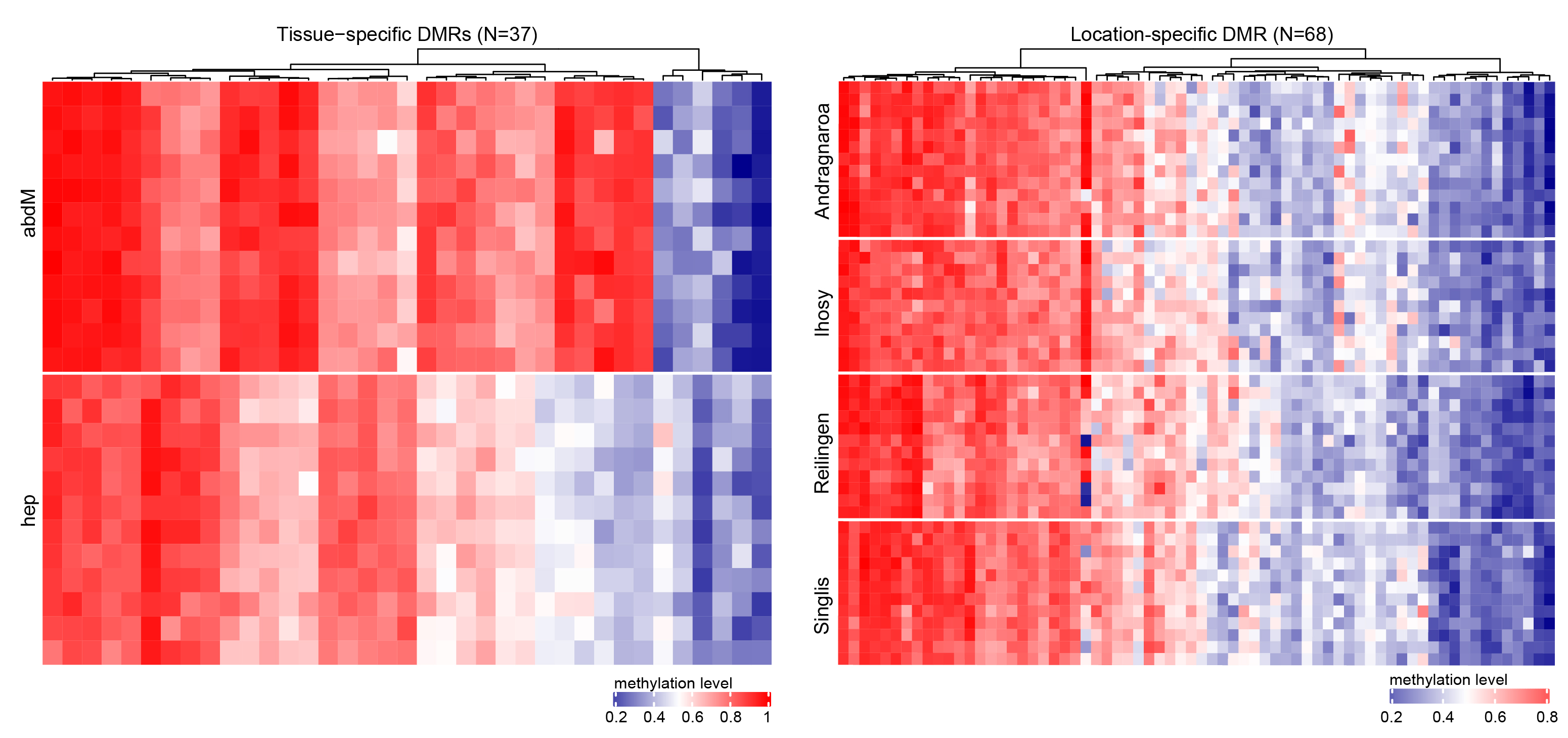
**

**Supplementary Figure S4.** Heatmaps showing average methylation levels of 37 tissue-specific and 68 location-specific DMRs. Methylation levels are indicated on a scale from 0 (blue) to 1 (red).

**Supplementary Table S1.** PCR primers used for validation experiments.

| **Name** | **Sequence** | **Amplicon length [bp]** |
| --- | --- | --- |
| T173_fwd | \| tcgtcggcagcgtcagatgtgtataagagacagGAATTATTTTATTTGTGATATTTTTTTAAT \| \| --- \| | 306 |
| T173_rev | \| gtctcgtgggctcggagatgtgtataagagacagATTAATCCACATAATATTTCACCAC \| \| --- \| |  |
| T595_fwd | \| tcgtcggcagcgtcagatgtgtataagagacagTGGAGATAAGTTAGTTTAATTAGGTTATAT \| \| --- \| | 348 |
| T595_rev | \| gtctcgtgggctcggagatgtgtataagagacagAATCATCTTAAAAATTCAAAAAAAA \| \| --- \| |  |
| L460_fwd | \| tcgtcggcagcgtcagatgtgtataagagacagGGGTAGATAGAATTATTTTTTTT \| \| --- \| | 184 |
| L460_rev | \| gtctcgtgggctcggagatgtgtataagagacagTTTCCTAAAAACCACATTAAAACAC \| \| --- \| |  |
| L88_R1_fwd | \| tcgtcggcagcgtcagatgtgtataagagacagTTATAATATATTAATGGTTTTGATGA \| \| --- \| | 284 |
| L88_R1_rev | \| gtctcgtgggctcggagatgtgtataagagacagCACAAAAAACAAAAACTACAAACTC \| \| --- \| |  |
| L88_R2_fwd | \| tcgtcggcagcgtcagatgtgtataagagacagATTATATTTATATTGGATGGATTTAATTTA \| \| --- \| | 126 |
| L88_R2_rev | \| gtctcgtgggctcggagatgtgtataagagacagAAACAAACATCTTATACAATTCTTCTC \| \| --- \| |  |

Names with T and L refer to tissue-specific and location-specific target genes, respectively. Sequences in red and blue represent the adapters required for the MiSeq platform.

**Supplementary Table S2.** List of samples with numbers of mapped sequencing reads.

| **Animal** | **Hepatopancreas**  **PE reads** | **Bisulfite conversion rate** | **Abdominal muscle**  **PE reads** | **Bisulfite conversion rate** |
| --- | --- | --- | --- | --- |
| Singlis 1 | 16,422,317 | 1.00 | 39,455,686 | 0.99 |
| Singlis 2 | 19,008,747 | 1.00 | 43,297,738 | 0.99 |
| Singlis 3 | 16,497,929 | 0.99 | 16,497,932 | 1.00 |
| Singlis 4 | 16,497,930 | 0.99 | 16,497,933 | 0.99 |
| Singlis 5 | 16,497,931 | 0.99 | not analyzed | not analyzed |
| Singlis 11 | 16,497,948 | 0.97 | 16,497,947 | 0.99 |
| Singlis 12 | 16,497,950 | 1.00 | 16,497,949 | 0.99 |
| Singlis 13 | 16,497,952 | 1.00 | 16,497,951 | 1.00 |
| Singlis 15 | 16,497,953 | 0.98 | 23,644,030 | 0.99 |
| Singlis 17 | 21,318,851 | 1.00 | 20,990,725 | 1.00 |
| Singlis 19 | 19,209,360 | 1.00 | 17,393,362 | 1.00 |
| Singlis 22 | 16,775,663 | 1.00 | 22,234,064 | 0.99 |
| Singlis 23 | not analyzed | not analyzed | 18,799,381 | 1.00 |
| Reilingen 1a | 15,636,129 | 0.99 | not analyzed | not analyzed |
| Reilingen 2a | 18,647,559 | 0.99 | not analyzed | not analyzed |
| Reilingen 3a | 17,627,673 | 0.98 | not analyzed | not analyzed |
| Reilingen 9 | 16,497,934 | 0.98 | 41,340,247 | 0.99 |
| Reilingen 10 | 13,795,480 | 0.99 | 50,614,390 | 0.99 |
| Reilingen 11 | not analyzed | not analyzed | 56,608,999 | 0.99 |
| Reilingen 12 | 12,500,600 | 0.99 | not analyzed | not analyzed |
| Reilingen 13 | 11,847,473 | 1.00 | not analyzed | not analyzed |
| Reilingen 14 | 16,466,402 | 0.99 | not analyzed | not analyzed |
| Reilingen 16 | 16,497,935 | 1.00 | not analyzed | not analyzed |
| Reilingen 20 | 16,497,936 | 0.98 | 22,342,003 | 0.99 |
| Reilingen 21 | 16,497,937 | 0.98 | 15,694,472 | 1.00 |
| Reilingen 23 | 16,497,938 | 0.99 | 19,405,527 | 1.00 |
| Andragnaroa 1 | 9,385,147 | 0.97 | not analyzed | not analyzed |
| Andragnaroa 3 | 20,804,742 | 0.98 | not analyzed | not analyzed |
| Andragnaroa 4 | 16,497,954 | 0.99 | 16,497,955 | 0.98 |
| Andragnaroa 5 | 14,450,800 | 0.94 | not analyzed | not analyzed |
| Andragnaroa 7 | 17,376,478 | 0.99 | not analyzed | not analyzed |
| Andragnaroa 9 | 21,217,303 | 0.98 | not analyzed | not analyzed |
| Andragnaroa 13 | 24,314,596 | 0.98 | 14,204,161 | 0.99 |
| Andragnaroa 14 | 25,805,349 | 0.99 | not analyzed | not analyzed |
| Andragnaroa 15 | 25,199,732 | 0.99 | 18,377,577 | 0.97 |
| Andragnaroa 41 | 20,452,025 | 1.00 | not analyzed | not analyzed |
| Andragnaroa 42 | 18,601,040 | 1.00 | not analyzed | not analyzed |
| Andragnaroa 44 | 16,414,095 | 0.98 | 32,665,345 | 0.99 |
| Ihosy 4 | 16,497,940 | 1.00 | 16,497,939 | 0.98 |
| Ihosy 5 | 10,984,213 | 1.00 | not analyzed | not analyzed |
| Ihosy 12 | 16,497,942 | 1.00 | 16,497,941 | 0.98 |
| Ihosy 16 | 15,801,536 | 0.99 | not analyzed | not analyzed |
| Ihosy 18 | 14,239,775 | 0.98 | not analyzed | not analyzed |
| Ihosy 27 | 16,497,944 | 1.00 | 16,497,943 | 0.99 |
| Ihosy 36 | 16,605,394 | 0.99 | not analyzed | not analyzed |
| Ihosy 37 | 15,478,965 | 0.99 | not analyzed | not analyzed |
| Ihosy 39 | 19,701,720 | 0.96 | not analyzed | not analyzed |
| Ihosy 43 | 17,922,783 | 0.99 | not analyzed | not analyzed |
| Ihosy 48 | 16,497,946 | 0.95 | 16,497,945 | 0.99 |

**Supplementary Table S3.** Core set of 361 variably methylated genes**.**

| **Gene ID** | **Scaffold** | **Start** | **End** |
| --- | --- | --- | --- |
| maker-scaffold304068-snap-gene-0.0 | scaffold304068 | 1337 | 27574 |
| snap_masked-scaffold24197-processed-gene-0.0 | scaffold24197 | 8904 | 43369 |
| snap-scaffold36687-processed-gene-0.8 | scaffold36687 | 137868 | 162515 |
| snap_masked-scaffold90387-processed-gene-0.16 | scaffold90387 | 50002 | 65769 |
| evm-scaffold108432-processed-gene-0.3 | scaffold108432 | 65051 | 76801 |
| evm-scaffold139595-processed-gene-0.11 | scaffold139595 | 4000 | 19145 |
| snap-scaffold26860-processed-gene-0.5 | scaffold26860 | 113376 | 137381 |
| evm-scaffold16904-processed-gene-1.0 | scaffold16904 | 183886 | 196760 |
| maker-scaffold10264-snap-gene-0.18 | scaffold10264 | 25066 | 37578 |
| maker-scaffold9659-snap-gene-1.19 | scaffold9659 | 203904 | 211046 |
| maker-scaffold2381-snap-gene-1.5 | scaffold2381 | 83970 | 96356 |
| evm-scaffold50337-processed-gene-0.4 | scaffold50337 | 54275 | 66946 |
| maker-scaffold45362-snap-gene-0.0 | scaffold45362 | 65031 | 78444 |
| maker-scaffold115264-snap-gene-0.3 | scaffold115264 | 19872 | 31054 |
| maker-scaffold10188-snap-gene-0.1 | scaffold10188 | 54147 | 60918 |
| snap_masked-scaffold50797-processed-gene-0.7 | scaffold50797 | 37447 | 42476 |
| snap-scaffold115264-processed-gene-0.9 | scaffold115264 | 38152 | 63093 |
| maker-scaffold11552-snap-gene-2.41 | scaffold11552 | 256598 | 273594 |
| maker-scaffold126600-snap-gene-0.20 | scaffold126600 | 85747 | 92192 |
| evm-scaffold12945-processed-gene-0.21 | scaffold12945 | 14168 | 20265 |
| snap_masked-scaffold93376-processed-gene-0.9 | scaffold93376 | 16276 | 32089 |
| maker-scaffold219941-snap-gene-0.1 | scaffold219941 | 2898 | 11055 |
| maker-scaffold15530-snap-gene-0.12 | scaffold15530 | 70666 | 87866 |
| maker-scaffold12744-snap-gene-1.27 | scaffold12744 | 114212 | 127348 |
| maker-scaffold8191-snap-gene-0.0 | scaffold8191 | 48342 | 67985 |
| maker-scaffold175420-snap-gene-0.0 | scaffold175420 | 16768 | 32937 |
| evm-scaffold112413-processed-gene-0.17 | scaffold112413 | 25163 | 31291 |
| snap-scaffold39846-processed-gene-0.9 | scaffold39846 | 18870 | 30259 |
| maker-scaffold121213-snap-gene-0.1 | scaffold121213 | 30065 | 35437 |
| snap_masked-scaffold43456-processed-gene-0.8 | scaffold43456 | 30046 | 39826 |
| maker-scaffold17132-snap-gene-0.32 | scaffold17132 | 3351 | 27102 |
| maker-scaffold267215-snap-gene-0.0 | scaffold267215 | 7481 | 13107 |
| maker-scaffold205616-snap-gene-0.0 | scaffold205616 | 49312 | 53787 |
| snap-scaffold53412-processed-gene-0.5 | scaffold53412 | 59522 | 68472 |
| maker-scaffold135435-snap-gene-0.1 | scaffold135435 | 249 | 9302 |
| snap-scaffold4868-processed-gene-0.30 | scaffold4868 | 36318 | 50961 |
| evm-scaffold41057-processed-gene-0.1 | scaffold41057 | 28601 | 33526 |
| maker-scaffold102285-snap-gene-0.10 | scaffold102285 | 38482 | 46524 |
| maker-scaffold220173-snap-gene-0.0 | scaffold220173 | 1241 | 9258 |
| maker-scaffold91737-snap-gene-0.0 | scaffold91737 | 39280 | 44975 |
| maker-scaffold6474-snap-gene-0.6 | scaffold6474 | 33723 | 47661 |
| evm-scaffold33165-processed-gene-0.3 | scaffold33165 | 58807 | 65868 |
| snap-scaffold8703-processed-gene-0.1 | scaffold8703 | 39503 | 43579 |
| maker-scaffold48239-snap-gene-0.18 | scaffold48239 | 64621 | 72046 |
| maker-scaffold32877-snap-gene-0.1 | scaffold32877 | 8946 | 23196 |
| maker-scaffold1498-snap-gene-0.3 | scaffold1498 | 57051 | 67352 |
| evm-scaffold94418-processed-gene-0.14 | scaffold94418 | 53835 | 60225 |
| maker-scaffold13345-snap-gene-1.11 | scaffold13345 | 82911 | 91955 |
| snap_masked-scaffold74137-processed-gene-0.3 | scaffold74137 | 17995 | 21318 |
| maker-scaffold50170-snap-gene-0.19 | scaffold50170 | 34890 | 40929 |
| evm-scaffold43820-processed-gene-0.1 | scaffold43820 | 71976 | 78177 |
| evm-scaffold172683-processed-gene-0.3 | scaffold172683 | 67195 | 72070 |
| maker-scaffold263285-snap-gene-0.1 | scaffold263285 | 22636 | 31057 |
| maker-scaffold123276-snap-gene-0.16 | scaffold123276 | 48317 | 60296 |
| maker-scaffold113704-exonerate_est2genome-gene-0.17 | scaffold113704 | 682 | 1469 |
| maker-scaffold4620-snap-gene-0.26 | scaffold4620 | 11979 | 20871 |
| maker-scaffold7189-snap-gene-0.3 | scaffold7189 | 19816 | 28919 |
| evm-scaffold16727-processed-gene-0.11 | scaffold16727 | 63585 | 71191 |
| maker-scaffold12256-snap-gene-0.0 | scaffold12256 | 28180 | 36440 |
| evm-scaffold397263-processed-gene-0.0 | scaffold397263 | 26651 | 30566 |
| evm-scaffold9304-processed-gene-0.27 | scaffold9304 | 97512 | 103845 |
| maker-scaffold114487-snap-gene-0.3 | scaffold114487 | 141172 | 149611 |
| maker-scaffold48239-exonerate_est2genome-gene-0.1 | scaffold48239 | 72267 | 72884 |
| maker-scaffold10961-snap-gene-0.5 | scaffold10961 | 464 | 7461 |
| evm-scaffold100674-processed-gene-0.5 | scaffold100674 | 62519 | 66202 |
| evm-scaffold9911-processed-gene-0.23 | scaffold9911 | 57148 | 61973 |
| maker-scaffold101782-snap-gene-0.0 | scaffold101782 | 359 | 3823 |
| evm-scaffold5511-processed-gene-0.0 | scaffold5511 | 19862 | 25147 |
| snap_masked-scaffold310636-processed-gene-0.1 | scaffold310636 | 12641 | 14932 |
| maker-scaffold13666-snap-gene-0.25 | scaffold13666 | 93821 | 101729 |
| maker-scaffold38912-snap-gene-0.1 | scaffold38912 | 35958 | 42540 |
| maker-scaffold38310-snap-gene-0.19 | scaffold38310 | 26015 | 28730 |
| evm-scaffold6249-processed-gene-0.16 | scaffold6249 | 13015 | 18415 |
| maker-scaffold124456-snap-gene-0.10 | scaffold124456 | 40484 | 46419 |
| maker-scaffold12620-snap-gene-0.21 | scaffold12620 | 879 | 5599 |
| maker-scaffold48310-snap-gene-0.0 | scaffold48310 | 8226 | 11931 |
| evm-scaffold34440-processed-gene-0.36 | scaffold34440 | 83604 | 88687 |
| maker-scaffold71508-snap-gene-0.7 | scaffold71508 | 1687 | 7045 |
| snap-scaffold6152-processed-gene-0.21 | scaffold6152 | 110089 | 114729 |
| maker-scaffold52598-snap-gene-0.3 | scaffold52598 | 4758 | 12239 |
| maker-scaffold54060-exonerate_est2genome-gene-0.2 | scaffold54060 | 7844 | 12054 |
| evm-scaffold39916-processed-gene-0.41 | scaffold39916 | 152669 | 158190 |
| maker-scaffold9999-snap-gene-0.39 | scaffold9999 | 123755 | 131121 |
| snap-scaffold14680-processed-gene-0.21 | scaffold14680 | 76788 | 82577 |
| maker-scaffold28267-snap-gene-0.0 | scaffold28267 | 7743 | 13738 |
| maker-scaffold394459-snap-gene-0.5 | scaffold394459 | 1518 | 8604 |
| evm-scaffold90817-processed-gene-0.1 | scaffold90817 | 9485 | 13683 |
| evm-scaffold371305-processed-gene-0.0 | scaffold371305 | 17158 | 21261 |
| maker-scaffold130709-exonerate_est2genome-gene-0.10 | scaffold130709 | 6192 | 13241 |
| maker-scaffold11851-snap-gene-0.5 | scaffold11851 | 77 | 5252 |
| maker-scaffold22339-snap-gene-0.0 | scaffold22339 | 1122 | 5657 |
| evm-scaffold107110-processed-gene-0.0 | scaffold107110 | 986 | 2634 |
| evm-scaffold73810-processed-gene-1.35 | scaffold73810 | 67198 | 69697 |
| evm-scaffold40617-processed-gene-0.7 | scaffold40617 | 42743 | 47819 |
| evm-scaffold137559-processed-gene-0.22 | scaffold137559 | 63163 | 67788 |
| maker-scaffold202891-snap-gene-0.5 | scaffold202891 | 428 | 4466 |
| snap_masked-scaffold81770-processed-gene-0.17 | scaffold81770 | 87096 | 89144 |
| maker-scaffold27888-snap-gene-0.2 | scaffold27888 | 56636 | 64796 |
| maker-scaffold339-snap-gene-1.14 | scaffold339 | 182807 | 188079 |
| evm-scaffold7906-processed-gene-1.0 | scaffold7906 | 90914 | 96317 |
| maker-scaffold564-snap-gene-1.5 | scaffold564 | 110968 | 116601 |
| snap_masked-scaffold104332-processed-gene-0.1 | scaffold104332 | 7495 | 13716 |
| maker-scaffold5412-snap-gene-1.1 | scaffold5412 | 147667 | 150797 |
| maker-scaffold22213-snap-gene-0.22 | scaffold22213 | 60151 | 68877 |
| maker-scaffold26595-snap-gene-0.19 | scaffold26595 | 32853 | 44683 |
| maker-scaffold23087-snap-gene-0.10 | scaffold23087 | 20936 | 26723 |
| evm-scaffold80512-processed-gene-0.10 | scaffold80512 | 66725 | 75346 |
| maker-scaffold17930-snap-gene-0.0 | scaffold17930 | 74641 | 76992 |
| snap_masked-scaffold868-processed-gene-1.34 | scaffold868 | 141766 | 146382 |
| maker-scaffold6973-snap-gene-0.2 | scaffold6973 | 4987 | 7505 |
| maker-scaffold1857-snap-gene-1.34 | scaffold1857 | 83854 | 91724 |
| snap_masked-scaffold91879-processed-gene-0.2 | scaffold91879 | 17111 | 28264 |
| maker-scaffold386719-snap-gene-0.2 | scaffold386719 | 6768 | 11610 |
| snap-scaffold30198-processed-gene-0.4 | scaffold30198 | 998 | 6259 |
| maker-scaffold16863-snap-gene-0.12 | scaffold16863 | 10901 | 15377 |
| maker-scaffold80517-snap-gene-0.0 | scaffold80517 | 24051 | 29834 |
| evm-scaffold228228-processed-gene-0.1 | scaffold228228 | 48536 | 52576 |
| snap-scaffold102750-processed-gene-0.6 | scaffold102750 | 75430 | 82953 |
| evm-scaffold1978-processed-gene-0.5 | scaffold1978 | 22655 | 29497 |
| evm-scaffold36395-processed-gene-0.8 | scaffold36395 | 9144 | 14617 |
| evm-scaffold59094-processed-gene-0.23 | scaffold59094 | 68984 | 73308 |
| evm-scaffold48548-processed-gene-0.0 | scaffold48548 | 17748 | 20389 |
| maker-scaffold377919-snap-gene-0.0 | scaffold377919 | 34891 | 42885 |
| snap-scaffold74799-processed-gene-0.5 | scaffold74799 | 75543 | 76292 |
| evm-scaffold74849-processed-gene-1.29 | scaffold74849 | 177285 | 182531 |
| snap_masked-scaffold59159-processed-gene-0.9 | scaffold59159 | 49876 | 50094 |
| snap_masked-scaffold2177-processed-gene-0.6 | scaffold2177 | 129902 | 135993 |
| evm-scaffold361614-processed-gene-0.1 | scaffold361614 | 8789 | 14371 |
| maker-scaffold81285-snap-gene-0.0 | scaffold81285 | 23168 | 25422 |
| maker-scaffold107280-snap-gene-0.0 | scaffold107280 | 19587 | 22364 |
| snap-scaffold111395-processed-gene-0.7 | scaffold111395 | 39120 | 45694 |
| maker-scaffold4989-snap-gene-0.21 | scaffold4989 | 47361 | 52650 |
| snap-scaffold61385-processed-gene-0.6 | scaffold61385 | 38072 | 39592 |
| evm-scaffold35783-processed-gene-0.1 | scaffold35783 | 25675 | 32243 |
| maker-scaffold50170-exonerate_est2genome-gene-0.0 | scaffold50170 | 33956 | 34825 |
| maker-scaffold38451-snap-gene-0.0 | scaffold38451 | 38756 | 45073 |
| snap_masked-scaffold25208-processed-gene-0.0 | scaffold25208 | 12 | 486 |
| maker-scaffold138460-exonerate_est2genome-gene-0.45 | scaffold138460 | 111216 | 111777 |
| snap-scaffold53368-processed-gene-0.1 | scaffold53368 | 11351 | 12349 |
| snap-scaffold16922-processed-gene-0.14 | scaffold16922 | 144576 | 147649 |
| maker-scaffold3650-snap-gene-0.0 | scaffold3650 | 51947 | 56482 |
| maker-scaffold112453-snap-gene-0.2 | scaffold112453 | 94164 | 97264 |
| maker-scaffold41290-snap-gene-2.1 | scaffold41290 | 227621 | 232155 |
| maker-scaffold10925-exonerate_est2genome-gene-0.28 | scaffold10925 | 43088 | 44269 |
| maker-scaffold3354-snap-gene-0.1 | scaffold3354 | 14246 | 19146 |
| snap-scaffold45749-processed-gene-0.6 | scaffold45749 | 28428 | 31630 |
| snap-scaffold81425-processed-gene-0.9 | scaffold81425 | 26428 | 35106 |
| maker-scaffold23229-snap-gene-1.15 | scaffold23229 | 109617 | 113443 |
| maker-scaffold73264-snap-gene-0.0 | scaffold73264 | 6157 | 8104 |
| snap_masked-scaffold62530-processed-gene-0.4 | scaffold62530 | 16714 | 18750 |
| snap-scaffold5751-processed-gene-0.4 | scaffold5751 | 29224 | 29448 |
| maker-scaffold59094-snap-gene-0.22 | scaffold59094 | 85362 | 87038 |
| maker-scaffold211263-snap-gene-0.11 | scaffold211263 | 40503 | 43319 |
| maker-scaffold25493-snap-gene-0.48 | scaffold25493 | 33080 | 37341 |
| maker-scaffold76097-snap-gene-0.13 | scaffold76097 | 61195 | 63396 |
| maker-scaffold1180-snap-gene-0.9 | scaffold1180 | 72593 | 78002 |
| maker-scaffold31717-snap-gene-0.2 | scaffold31717 | 60581 | 68418 |
| maker-scaffold44746-snap-gene-0.0 | scaffold44746 | 66445 | 71453 |
| evm-scaffold22394-processed-gene-2.5 | scaffold22394 | 251018 | 254621 |
| snap_masked-scaffold9798-processed-gene-0.0 | scaffold9798 | 21268 | 21624 |
| maker-scaffold215670-snap-gene-0.0 | scaffold215670 | 5627 | 11303 |
| maker-scaffold21855-snap-gene-0.4 | scaffold21855 | 132449 | 136040 |
| maker-scaffold61175-snap-gene-0.20 | scaffold61175 | 47087 | 48344 |
| snap_masked-scaffold5220-processed-gene-1.12 | scaffold5220 | 154619 | 155515 |
| maker-scaffold72239-snap-gene-0.8 | scaffold72239 | 4943 | 8293 |
| snap-scaffold27036-processed-gene-0.0 | scaffold27036 | 18815 | 19618 |
| snap-scaffold122449-processed-gene-0.0 | scaffold122449 | 1099 | 1506 |
| maker-scaffold41290-snap-gene-1.0 | scaffold41290 | 94934 | 98362 |
| maker-scaffold156213-snap-gene-1.20 | scaffold156213 | 106417 | 108341 |
| maker-scaffold39916-snap-gene-0.48 | scaffold39916 | 147719 | 152559 |
| snap-scaffold1620-processed-gene-1.39 | scaffold1620 | 229567 | 233057 |
| maker-scaffold10917-snap-gene-0.1 | scaffold10917 | 99892 | 101179 |
| evm-scaffold39916-processed-gene-0.39 | scaffold39916 | 115273 | 119446 |
| maker-scaffold8594-snap-gene-0.3 | scaffold8594 | 161003 | 165873 |
| maker-scaffold156352-snap-gene-0.0 | scaffold156352 | 4759 | 8791 |
| maker-scaffold262363-snap-gene-0.0 | scaffold262363 | 25460 | 29529 |
| snap_masked-scaffold41199-processed-gene-0.3 | scaffold41199 | 28695 | 29186 |
| maker-scaffold2625-exonerate_est2genome-gene-1.48 | scaffold2625 | 169586 | 173199 |
| snap-scaffold135378-processed-gene-0.13 | scaffold135378 | 80922 | 85145 |
| evm-scaffold9975-processed-gene-1.28 | scaffold9975 | 92463 | 98507 |
| snap-scaffold135539-processed-gene-0.4 | scaffold135539 | 36766 | 37365 |
| snap-scaffold70321-processed-gene-0.9 | scaffold70321 | 72790 | 73173 |
| evm-scaffold56737-processed-gene-0.25 | scaffold56737 | 33595 | 36872 |
| evm-scaffold49405-processed-gene-0.2 | scaffold49405 | 57239 | 60293 |
| snap_masked-scaffold19330-processed-gene-0.11 | scaffold19330 | 46109 | 46777 |
| snap_masked-scaffold23847-processed-gene-0.23 | scaffold23847 | 106662 | 107048 |
| snap-scaffold5583-processed-gene-1.21 | scaffold5583 | 141290 | 141757 |
| snap-scaffold5020-processed-gene-0.4 | scaffold5020 | 37952 | 38401 |
| snap-scaffold116111-processed-gene-0.3 | scaffold116111 | 14899 | 15399 |
| snap-scaffold7627-processed-gene-0.4 | scaffold7627 | 45053 | 45893 |
| snap-scaffold91170-processed-gene-0.1 | scaffold91170 | 764 | 1429 |
| maker-scaffold12911-snap-gene-0.5 | scaffold12911 | 69371 | 71899 |
| snap-scaffold352968-processed-gene-0.0 | scaffold352968 | 568 | 1035 |
| snap-scaffold19330-processed-gene-0.4 | scaffold19330 | 26274 | 28769 |
| snap-scaffold52698-processed-gene-0.12 | scaffold52698 | 39460 | 39846 |
| maker-scaffold16344-exonerate_est2genome-gene-0.22 | scaffold16344 | 54299 | 56148 |
| maker-scaffold18679-snap-gene-0.48 | scaffold18679 | 92344 | 92876 |
| snap-scaffold257007-processed-gene-0.6 | scaffold257007 | 27732 | 28088 |
| snap_masked-scaffold522-processed-gene-0.3 | scaffold522 | 50041 | 50616 |
| snap-scaffold5124-processed-gene-0.4 | scaffold5124 | 12695 | 12982 |
| maker-scaffold25095-snap-gene-0.69 | scaffold25095 | 63863 | 64998 |
| snap-scaffold32024-processed-gene-0.3 | scaffold32024 | 24648 | 24866 |
| evm-scaffold83705-processed-gene-0.1 | scaffold83705 | 25046 | 28714 |
| evm-scaffold134054-processed-gene-0.11 | scaffold134054 | 29553 | 32804 |
| evm-scaffold57-processed-gene-1.48 | scaffold57 | 104482 | 108289 |
| snap-scaffold52598-processed-gene-0.25 | scaffold52598 | 107050 | 107586 |
| snap-scaffold21794-processed-gene-0.26 | scaffold21794 | 69850 | 70434 |
| snap_masked-scaffold22145-processed-gene-0.1 | scaffold22145 | 688 | 954 |
| snap_masked-scaffold87134-processed-gene-0.3 | scaffold87134 | 23056 | 23358 |
| snap-scaffold54195-processed-gene-0.39 | scaffold54195 | 98175 | 98477 |
| snap_masked-scaffold18008-processed-gene-0.1 | scaffold18008 | 19654 | 20070 |
| maker-scaffold333883-exonerate_est2genome-gene-0.0 | scaffold333883 | 9208 | 9684 |
| snap_masked-scaffold140642-processed-gene-0.7 | scaffold140642 | 10935 | 11473 |
| maker-scaffold140642-exonerate_est2genome-gene-0.0 | scaffold140642 | 11139 | 11740 |
| evm-scaffold10046-processed-gene-0.0 | scaffold10046 | 61937 | 64677 |
| maker-scaffold11617-snap-gene-0.34 | scaffold11617 | 27592 | 31834 |
| snap-scaffold140713-processed-gene-0.3 | scaffold140713 | 31608 | 38022 |
| snap_masked-scaffold98835-processed-gene-0.5 | scaffold98835 | 34867 | 35255 |
| snap-scaffold35469-processed-gene-0.3 | scaffold35469 | 36010 | 36411 |
| maker-scaffold117568-exonerate_est2genome-gene-0.7 | scaffold117568 | 15868 | 16247 |
| evm-scaffold742-processed-gene-0.36 | scaffold742 | 61057 | 63185 |
| evm-scaffold4470-processed-gene-1.4 | scaffold4470 | 120489 | 122455 |
| maker-scaffold46239-snap-gene-0.1 | scaffold46239 | 87878 | 90794 |
| snap-scaffold3259-processed-gene-1.3 | scaffold3259 | 50485 | 50827 |
| snap-scaffold317362-processed-gene-0.1 | scaffold317362 | 1192 | 1482 |
| snap-scaffold10188-processed-gene-0.18 | scaffold10188 | 27890 | 29985 |
| snap-scaffold122226-processed-gene-0.3 | scaffold122226 | 40393 | 40945 |
| snap-scaffold50170-processed-gene-0.7 | scaffold50170 | 1950 | 2341 |
| snap_masked-scaffold207763-processed-gene-0.2 | scaffold207763 | 17887 | 18698 |
| snap_masked-scaffold92118-processed-gene-0.3 | scaffold92118 | 11370 | 11660 |
| snap-scaffold168208-processed-gene-0.0 | scaffold168208 | 855 | 1424 |
| maker-scaffold134109-snap-gene-0.14 | scaffold134109 | 39275 | 41980 |
| maker-scaffold6421-snap-gene-0.31 | scaffold6421 | 36942 | 39630 |
| maker-scaffold60601-exonerate_est2genome-gene-0.20 | scaffold60601 | 11934 | 12862 |
| maker-scaffold97830-snap-gene-0.2 | scaffold97830 | 18417 | 18937 |
| snap-scaffold5315-processed-gene-0.29 | scaffold5315 | 45483 | 45707 |
| snap-scaffold28753-processed-gene-0.18 | scaffold28753 | 78018 | 78470 |
| snap_masked-scaffold367392-processed-gene-0.11 | scaffold367392 | 7787 | 8014 |
| snap-scaffold49466-processed-gene-0.4 | scaffold49466 | 2519 | 2848 |
| snap-scaffold392560-processed-gene-0.4 | scaffold392560 | 11902 | 12204 |
| snap-scaffold15934-processed-gene-0.3 | scaffold15934 | 149781 | 150110 |
| snap_masked-scaffold18992-processed-gene-0.6 | scaffold18992 | 46014 | 46271 |
| snap_masked-scaffold146957-processed-gene-0.3 | scaffold146957 | 26384 | 27918 |
| snap-scaffold25878-processed-gene-0.9 | scaffold25878 | 15107 | 15409 |
| snap_masked-scaffold73424-processed-gene-0.1 | scaffold73424 | 7297 | 7599 |
| snap_masked-scaffold97644-processed-gene-0.15 | scaffold97644 | 10259 | 10567 |
| snap_masked-scaffold53654-processed-gene-0.3 | scaffold53654 | 7191 | 7771 |
| maker-scaffold47681-exonerate_est2genome-gene-0.0 | scaffold47681 | 356 | 970 |
| maker-scaffold31708-snap-gene-0.2 | scaffold31708 | 69163 | 73176 |
| maker-scaffold6368-snap-gene-0.42 | scaffold6368 | 101857 | 106342 |
| snap-scaffold75609-processed-gene-0.2 | scaffold75609 | 6101 | 11966 |
| snap_masked-scaffold225859-processed-gene-0.4 | scaffold225859 | 45899 | 46424 |
| snap-scaffold25619-processed-gene-0.14 | scaffold25619 | 11173 | 11799 |
| evm-scaffold13441-processed-gene-0.0 | scaffold13441 | 117539 | 120929 |
| snap_masked-scaffold22208-processed-gene-1.23 | scaffold22208 | 130498 | 130764 |
| snap-scaffold90609-processed-gene-0.36 | scaffold90609 | 47019 | 47240 |
| snap-scaffold157241-processed-gene-0.8 | scaffold157241 | 35342 | 35566 |
| snap_masked-scaffold54060-processed-gene-0.3 | scaffold54060 | 2684 | 3304 |
| snap_masked-scaffold195460-processed-gene-0.3 | scaffold195460 | 39668 | 40474 |
| snap_masked-scaffold10502-processed-gene-0.7 | scaffold10502 | 12267 | 12569 |
| snap_masked-scaffold142074-processed-gene-0.0 | scaffold142074 | 20258 | 20557 |
| snap_masked-scaffold43914-processed-gene-0.1 | scaffold43914 | 42702 | 43364 |
| maker-scaffold16651-exonerate_est2genome-gene-0.0 | scaffold16651 | 73734 | 74441 |
| maker-scaffold44294-exonerate_est2genome-gene-0.1 | scaffold44294 | 896 | 1512 |
| snap-scaffold37344-processed-gene-0.10 | scaffold37344 | 77552 | 78040 |
| snap-scaffold23679-processed-gene-1.15 | scaffold23679 | 210879 | 211460 |
| snap-scaffold5808-processed-gene-1.32 | scaffold5808 | 182568 | 182987 |
| evm-scaffold22787-processed-gene-0.15 | scaffold22787 | 53527 | 53951 |
| snap-scaffold17307-processed-gene-0.2 | scaffold17307 | 2378 | 2863 |
| maker-scaffold7189-exonerate_est2genome-gene-0.9 | scaffold7189 | 88683 | 89274 |
| maker-scaffold43849-exonerate_est2genome-gene-0.19 | scaffold43849 | 61106 | 63365 |
| snap_masked-scaffold61451-processed-gene-0.2 | scaffold61451 | 8144 | 8368 |
| snap-scaffold26326-processed-gene-0.0 | scaffold26326 | 965 | 1421 |
| snap-scaffold182519-processed-gene-0.1 | scaffold182519 | 6486 | 6770 |
| snap_masked-scaffold9248-processed-gene-0.0 | scaffold9248 | 7599 | 8186 |
| maker-scaffold42144-snap-gene-0.3 | scaffold42144 | 68485 | 69224 |
| maker-scaffold30907-exonerate_est2genome-gene-0.43 | scaffold30907 | 78759 | 79432 |
| snap_masked-scaffold12875-processed-gene-0.20 | scaffold12875 | 106918 | 107486 |
| snap_masked-scaffold318945-processed-gene-0.0 | scaffold318945 | 16777 | 17068 |
| snap-scaffold114005-processed-gene-0.6 | scaffold114005 | 6959 | 7234 |
| snap-scaffold5655-processed-gene-0.6 | scaffold5655 | 49042 | 49332 |
| snap-scaffold53979-processed-gene-0.5 | scaffold53979 | 9617 | 9799 |
| evm-scaffold96038-processed-gene-0.1 | scaffold96038 | 71623 | 72027 |
| snap-scaffold120289-processed-gene-0.3 | scaffold120289 | 15738 | 15929 |
| maker-scaffold597-snap-gene-0.30 | scaffold597 | 94782 | 98489 |
| maker-scaffold135148-exonerate_est2genome-gene-0.9 | scaffold135148 | 37858 | 38972 |
| maker-scaffold112101-snap-gene-0.0 | scaffold112101 | 558 | 4634 |
| snap-scaffold17754-processed-gene-0.6 | scaffold17754 | 41594 | 42108 |
| snap-scaffold66720-processed-gene-0.28 | scaffold66720 | 47972 | 48286 |
| snap-scaffold23880-processed-gene-0.19 | scaffold23880 | 145666 | 146250 |
| maker-scaffold154965-snap-gene-0.18 | scaffold154965 | 19696 | 21012 |
| maker-scaffold5618-exonerate_est2genome-gene-0.26 | scaffold5618 | 111062 | 111528 |
| maker-scaffold27133-snap-gene-0.30 | scaffold27133 | 50671 | 52849 |
| snap-scaffold51555-processed-gene-0.24 | scaffold51555 | 110439 | 110771 |
| evm-scaffold89004-processed-gene-0.12 | scaffold89004 | 40733 | 41542 |
| snap_masked-scaffold25641-processed-gene-0.2 | scaffold25641 | 81893 | 82177 |
| snap-scaffold29669-processed-gene-0.4 | scaffold29669 | 70525 | 70887 |
| evm-scaffold112453-processed-gene-0.6 | scaffold112453 | 84131 | 86775 |
| snap-scaffold9956-processed-gene-0.2 | scaffold9956 | 13943 | 15844 |
| snap_masked-scaffold149691-processed-gene-0.6 | scaffold149691 | 13775 | 14008 |
| snap_masked-scaffold15951-processed-gene-0.3 | scaffold15951 | 66902 | 67192 |
| maker-scaffold17870-snap-gene-0.0 | scaffold17870 | 21506 | 22472 |
| snap_masked-scaffold5888-processed-gene-0.0 | scaffold5888 | 18203 | 19313 |
| maker-scaffold96861-exonerate_est2genome-gene-0.48 | scaffold96861 | 91008 | 92647 |
| maker-scaffold75304-snap-gene-0.8 | scaffold75304 | 32568 | 39530 |
| maker-scaffold85799-exonerate_est2genome-gene-0.3 | scaffold85799 | 44744 | 45723 |
| snap_masked-scaffold7926-processed-gene-1.11 | scaffold7926 | 174259 | 174552 |
| maker-scaffold41486-exonerate_est2genome-gene-0.21 | scaffold41486 | 72418 | 72877 |
| snap-scaffold16694-processed-gene-0.28 | scaffold16694 | 128439 | 128801 |
| snap_masked-scaffold27023-processed-gene-0.7 | scaffold27023 | 6270 | 6638 |
| snap-scaffold149077-processed-gene-0.6 | scaffold149077 | 17024 | 17338 |
| snap_masked-scaffold1389-processed-gene-0.12 | scaffold1389 | 187934 | 188233 |
| snap_masked-scaffold37805-processed-gene-0.26 | scaffold37805 | 75715 | 76116 |
| evm-scaffold60124-processed-gene-0.2 | scaffold60124 | 60398 | 60652 |
| snap-scaffold126287-processed-gene-0.21 | scaffold126287 | 44902 | 45132 |
| maker-scaffold15699-exonerate_est2genome-gene-0.11 | scaffold15699 | 34204 | 34719 |
| maker-scaffold131190-exonerate_est2genome-gene-0.9 | scaffold131190 | 6849 | 7378 |
| snap_masked-scaffold383077-processed-gene-0.1 | scaffold383077 | 17378 | 20322 |
| snap-scaffold113751-processed-gene-0.3 | scaffold113751 | 56577 | 56928 |
| snap-scaffold14417-processed-gene-0.23 | scaffold14417 | 35495 | 35719 |
| snap_masked-scaffold143691-processed-gene-0.0 | scaffold143691 | 17167 | 17457 |
| snap-scaffold22024-processed-gene-0.11 | scaffold22024 | 7267 | 7887 |
| snap_masked-scaffold281786-processed-gene-0.0 | scaffold281786 | 22200 | 22643 |
| snap_masked-scaffold49405-processed-gene-0.7 | scaffold49405 | 30954 | 31334 |
| snap_masked-scaffold8695-processed-gene-0.15 | scaffold8695 | 37705 | 38252 |
| snap_masked-scaffold38140-processed-gene-1.16 | scaffold38140 | 150406 | 150717 |
| snap-scaffold59103-processed-gene-0.6 | scaffold59103 | 48886 | 49305 |
| snap_masked-scaffold124521-processed-gene-0.0 | scaffold124521 | 373 | 759 |
| snap-scaffold44955-processed-gene-1.3 | scaffold44955 | 101327 | 101593 |
| maker-scaffold19557-exonerate_est2genome-gene-0.9 | scaffold19557 | 6375 | 7006 |
| snap-scaffold63049-processed-gene-0.6 | scaffold63049 | 6898 | 7185 |
| snap-scaffold12681-processed-gene-0.34 | scaffold12681 | 137021 | 137359 |
| snap-scaffold100333-processed-gene-0.7 | scaffold100333 | 68078 | 68435 |
| snap-scaffold132283-processed-gene-0.9 | scaffold132283 | 14227 | 14598 |
| maker-scaffold23128-exonerate_est2genome-gene-0.0 | scaffold23128 | 55624 | 56855 |
| snap-scaffold49585-processed-gene-0.9 | scaffold49585 | 39805 | 40749 |
| snap_masked-scaffold170217-processed-gene-0.6 | scaffold170217 | 284 | 832 |
| snap_masked-scaffold4828-processed-gene-0.20 | scaffold4828 | 80125 | 80586 |
| snap-scaffold165790-processed-gene-0.12 | scaffold165790 | 21438 | 21743 |
| snap-scaffold72681-processed-gene-0.14 | scaffold72681 | 2228 | 2557 |
| snap-scaffold13217-processed-gene-1.9 | scaffold13217 | 152763 | 153143 |
| snap_masked-scaffold112526-processed-gene-0.1 | scaffold112526 | 5342 | 5608 |
| snap_masked-scaffold126021-processed-gene-0.0 | scaffold126021 | 237 | 743 |
| snap-scaffold26866-processed-gene-0.8 | scaffold26866 | 17201 | 17425 |
| snap-scaffold15883-processed-gene-0.11 | scaffold15883 | 89609 | 89926 |
| snap-scaffold154958-processed-gene-0.7 | scaffold154958 | 44798 | 45049 |
| maker-scaffold85799-exonerate_est2genome-gene-0.0 | scaffold85799 | 2818 | 3674 |
| maker-scaffold49466-exonerate_est2genome-gene-0.1 | scaffold49466 | 3277 | 4209 |
| snap_masked-scaffold70663-processed-gene-0.1 | scaffold70663 | 15650 | 16044 |
| snap_masked-scaffold161560-processed-gene-0.0 | scaffold161560 | 44177 | 44662 |
| snap_masked-scaffold2950-processed-gene-0.0 | scaffold2950 | 11829 | 12179 |
| snap-scaffold285703-processed-gene-0.0 | scaffold285703 | 87 | 635 |
| maker-scaffold76455-exonerate_est2genome-gene-0.2 | scaffold76455 | 42725 | 43264 |
| snap_masked-scaffold106759-processed-gene-0.11 | scaffold106759 | 12108 | 12389 |
| snap-scaffold129183-processed-gene-0.1 | scaffold129183 | 9039 | 9380 |
| snap-scaffold2393-processed-gene-0.34 | scaffold2393 | 49989 | 50330 |
| snap-scaffold185801-processed-gene-0.10 | scaffold185801 | 126046 | 126426 |
| snap_masked-scaffold68245-processed-gene-0.4 | scaffold68245 | 303 | 719 |
| maker-scaffold270646-exonerate_est2genome-gene-0.0 | scaffold270646 | 2214 | 2653 |
| snap-scaffold315078-processed-gene-0.0 | scaffold315078 | 666 | 1793 |
| maker-scaffold13217-exonerate_est2genome-gene-1.53 | scaffold13217 | 203895 | 204872 |

Scaffold numbers are from the V0.4 genome assembly (Gutekunst et al., 2018).
